# Oral anticoagulants and risk of acute liver injury in patients with nonvalvular atrial fibrillation: a propensity-weighted nationwide cohort study

**DOI:** 10.1038/s41598-020-68304-8

**Published:** 2020-07-15

**Authors:** Géric Maura, Marc Bardou, Cécile Billionnet, Alain Weill, Jérôme Drouin, Anke Neumann

**Affiliations:** 1 French National Health Insurance (Caisse Nationale de L’Assurance Maladie, Cnam), 50 Avenue du Pr. André Lemierre, 75 986 Paris Cedex 20, France; 2 Clinical Investigation Center, Clinical Epidemiology/Clinical Trials Unit, Dijon-Bourgogne University Hospital, 21 000 Dijon, France; 3 Division of Gastroenterology, Dijon-Bourgogne University Hospital, 21 000 Dijon, France; 4 EPI-PHARE Epidemiology of Health Products, French National Agency for Medicines and Health Products Safety (ANSM) and French National Health Insurance (CNAM), 93 200, Saint-Denis, France

**Keywords:** Hepatology, Cardiology, Epidemiology

## Abstract

Insufficient real-world data on acute liver injury (ALI) risk associated with oral anticoagulants (OACs) exist in patients with nonvalvular atrial fibrillation (NVAF). Using the French national healthcare databases, a propensity-weighted nationwide cohort study was performed in NVAF patients initiating OACs from 2011 to 2016, considering separately those (1) with no prior liver disease (PLD) as main population, (2) with PLD, (3) with a history of chronic alcoholism. A Cox proportional hazards model was used to estimate the hazard ratio with 95% confidence interval (HR [95% CI]) of serious ALI (hospitalised ALI or liver transplantation) during the first year of treatment, for each non-vitamin K antagonist (VKA) oral anticoagulant (NOAC: dabigatran, rivaroxaban, apixaban) versus VKA. In patients with no PLD (N = 434,015), only rivaroxaban new users were at increased risk of serious ALI compared to VKA initiation (adjusted HR: 1.41 [1.05–1.91]). In patients with chronic alcoholism history (N = 13,173), only those initiating dabigatran were at increased risk of serious ALI compared to VKA (2.88 [1.74–4.76]) but an ancillary outcome suggested that differential clinical follow-up between groups might partly explain this association. In conclusion, this study does not suggest an increase of the 1-year risk of ALI in NOAC versus VKA patients with AF.

## Introduction

Non-vitamin K antagonist oral anticoagulants (NOACs) use has increased dramatically worldwide over the last 5 years since their introduction for stroke prevention in patients with nonvalvular atrial fibrillation (NVAF)^1^. The European guidelines now recommend NOACs in preference to vitamin K antagonists (VKAs) in these patients^2^. With increasing AF prevalence^3^ and, as a more convenient alternative to VKAs^4^, NOAC prescription should continue to increase. Careful monitoring of their safety profile apart from their well-known bleeding adverse events is therefore needed in clinical practice, especially regarding rare adverse events that cannot be observed in randomized clinical trials (RCT)^5^.

Following market withdrawal of ximelagatran, the first orally available direct thrombin inhibitor, due to reports of severe liver injury^6^, post-marketing data including pharmacovigilance reports have raised concerns about the potential hepatotoxicity of other NOACs^7–9^. While all NOACs undergo varying degrees of hepatic metabolism, with much lower hepatic metabolism for dabigatran^10^, cases of drug-induced liver injury, albeit rare and of varying severity, have been reported with dabigatran^11^, rivaroxaban^12^ and apixaban^13^. Although VKAs have been marketed for decades, acute liver failure has been rarely reported^14,15^. Only two published cohort studies based on claims data have compared NOAC and VKA therapies in terms of liver injuries^16,17^. Both studies defined liver injury as a mix of hospitalised acute and chronic (or unspecified) liver injuries and their estimates were based on small numbers of cases, leading to wide confidence intervals. Results of these observational studies contrasted with those reported from postmarketing pharmacovigilance data^18,19^. Furthermore, the risk of liver injury associated with oral anticoagulant (OAC) therapies has never been assessed in patients with concomitant AF and chronic alcoholism, which may concern about 1.9–7.5% of AF patients according to EORP-AF and EHS registry data^20,21^.

Due to the insufficient real-world data on liver injury risk associated with each type of oral anticoagulants to make informed choices in clinical practice, we therefore investigated the risk of serious acute liver injury (ALI) associated with OAC use and compared this risk between each NOAC (dabigatran, rivaroxaban, apixaban) and VKAs in AF patients, with no prior liver disease and also in those with prior liver disease and those with a history of chronic alcoholism.

## Methods

### Data sources

This observational study was conducted using data from the largest scheme (‘Régime général’, around 54 million beneficiaries) of the French national healthcare database (*Système National des Données de Santé,* SNDS). The SNDS database contains comprehensive data on health spending reimbursements and provides detailed medical information on all hospitalisations in France (see Supplementary Table 1). It has been previously described and used in pharmacoepidemiology research^22,23^, including in the field of gastrointestinal and liver diseases^24,25^.

### Study population and study design

First, a cohort of OAC new users was identified among patients with ‘Régime général’ health insurance coverage for at least 5 years (the calendar year of the index date and the previous 5 years) as those who initiated OAC therapy (all doses) with VKAs, dabigatran, rivaroxaban or apixaban (no reimbursement for any OAC, either VKA or NOAC, in the previous 24 months) between 1 January 2011 and 31 December 2016 (see design diagram in Supplementary Figure 1). A patient’s index date was the date of first OAC reimbursement during this period. The cohort of OAC new users was further restricted to those treated for NVAF: (1) patients treated for other OAC indications, i.e. patients with a history of deep vein thrombosis/pulmonary embolism (DVT/PE) or who had undergone lower limb orthopaedic procedures during the six-week pre-index period, were excluded; (2) OAC new users treated for AF were identified from the resulting cohort as the sum of “OAC new users with confirmed AF”, identified directly by using the International Classification of Diseases, 10th edition (ICD-10) I48 diagnosis code or specific AF management procedures, and “OAC new users with probable AF”, for outpatients identified using an algorithm discriminating AF from DVT/PE with 95% specificity^26^; (3) NVAF patients were identified by excluding patients with a history of prosthetic heart valve or chronic rheumatic heart disease.

Second, patients presenting contraindications to OAC therapy, currently or recently treated for cancer or treated for HIV infection, were excluded.

Finally, for the main analysis, the resulting cohort was restricted to patients with no history of liver disease during a 5-year pre-index period, including alcoholic liver disease, chronic hepatitis or disorders of copper or iron metabolism. Codes used for identification of the study population are shown in Supplementary Table 2.

#### Additional populations

Two additional study populations were defined from the incident cohort of OAC new users with NVAF: (1) patients with prior liver disease; (2) patients treated/hospitalised for chronic alcoholism in the 1-year rolling period before OAC initiation. The exact level of alcohol consumption and the patient’s history of alcoholism are not available in the French healthcare databases. Chronic alcoholism was therefore defined by using proxies (see definition in the Supplementary Table 2).

### Exposure

NOAC (dabigatran, rivaroxaban, apixaban) and VKA (fluindione, warfarin and acenocoumarol) therapies were identified using codes from the Anatomical Therapeutic Chemical (ATC) classification; edoxaban was not available during the period considered. A 30-day coverage period was attributed to any date of OAC reimbursement, including VKA.

### Outcome

The outcome of interest was serious ALI. Patients were considered to present this outcome when they had been hospitalised for liver transplantation (French procedure classification system, codes HLEA001, HLEA002) or presented one of the following ICD-10 diagnosis codes (considering only the principal discharge code): non-chronic toxic liver disease (K71.0, K71.1, K71.2, K71.6, K71.8, K71.9), non-chronic hepatic failure (K72.0, K72.9), nonspecific reactive hepatitis (K75.2), unspecified inflammatory liver disease (K75.9) or unspecified jaundice (R17). The use of these specific hospital codes, has been shown to maximize validity of ALI cases in a study based on four European healthcare data sources^27,28^. Only the first hospitalisation for ALI was considered during the follow-up period.

Regular International Normalised Ratio (INR) testing is performed in VKA patients, while no anticoagulation monitoring is needed in NOAC patients. Laboratory monitoring of toxic liver damage by transaminase assays (aspartate and alanine aminotransferases) was therefore used as an ancillary outcome to try to assess the potential role of differential anticoagulation blood monitoring in the early detection of ALI and to help to interpret results from cases of hospitalised ALI. As the French healthcare databases do not contain laboratory test results, a proxy for elevation of transaminases was used: a patient was considered to have elevation of transaminases when he/she presented consecutive reimbursements for serum transaminase assays (French laboratory test classification system codes 0.516, 0.517, 0.522) on at least three different dates over a 30-day period during follow-up.

### Covariates

Baseline covariates included sociodemographics, comorbidities, comedications and healthcare utilization proxies^29^, as listed in Table 1 and defined in Supplementary Table 2. In particular, a list of potential hepatotoxic drugs was considered at baseline^30,31^, including paracetamol, amoxicillin/clavulanic acid and rifampicin (see Supplementary Table 3).

**Table 1:** Baseline characteristics according to the type of OAC after inverse probability of treatment weighting in patients with no prior liver disease.

Characteristics (column %, unless stated otherwise)	VKA N = 220,367	Dabigatran N = 51,737	STD*	VKA N = 220,367	Rivaroxaban N = 99,408	STD*	VKA N = 220,367	Apixaban N = 62,503	STD*
**Sociodemographic data**									
Age (years), mean (SD)	76.3 (11.3)	76.6 (11.1)	0.03	75.7 (11.5)	76.1 (11.6)	0.04	76.4 (11.3)	76.9 (11.1)	0.04
18–54	4.5	4.0	− 0.02	5.0	4.6	− 0.02	4.4	3.8	− 0.03
55–64	10.6	10.2	− 0.01	11.2	10.6	− 0.02	10.4	9.8	− 0.02
65–74	22.0	21.7	− 0.01	23.3	22.5	− 0.02	22.1	21.5	− 0.02
75–79	17.1	16.9	− 0.01	17.1	16.9	− 0.01	16.8	16.9	0.00
80–84	20.8	21.2	0.01	20.0	20.3	0.01	20.8	21.0	0.01
85–89	16.5	17.1	0.02	15.4	16.1	0.02	16.7	17.5	0.02
≥ 90	8.5	8.9	0.01	7.9	9.0	0.04	8.8	9.5	0.02
Female sex	53.2	53.7	0.01	52.5	53.5	0.02	53.4	54.0	0.01
Deprivation index									
Quintile 1 (least deprived)	16.5	16.5	0.00	17.0	16.9	0.00	16.4	16.3	0.00
Quintile 2	18.5	18.6	0.00	19.0	18.9	0.00	18.8	18.6	− 0.01
Quintile 3	19.8	19.4	− 0.01	19.7	19.4	− 0.01	19.9	19.6	− 0.01
Quintile 4	21.2	21.2	0.00	20.9	21.0	0.00	21.2	21.4	0.01
Quintile 5 (most deprived)	22.4	22.7	0.01	22.0	22.3	0.01	22.3	22.6	0.01
Overseas departments	1.6	1.6	0.00	1.5	1.5	0.00	1.4	1.5	0.00
**Comorbidities**									
Ischaemic heart disease	28.4	28.7	0.01	27.1	27.5	0.01	28.4	28.6	0.00
Vascular disease	34.4	35.0	0.01	32.9	33.6	0.02	34.4	35.0	0.01
Heart failure	36.9	36.6	0.00	35.8	36.2	0.01	37.8	38.0	0.00
Arrhythmias (other than atrial fibrillation)†	22.7	23.0	0.01	21.9	22.4	0.01	22.6	23.3	0.02
Diabetes	23.9	24.4	0.01	23.3	23.6	0.01	24.0	24.0	0.00
History of arterial thromboembolic events†	14.6	15.5	0.03	13.4	14.4	0.03	14.6	15.4	0.02
Dementia or Parkinson’s disease	8.8	9.4	0.02	8.3	9.2	0.03	8.7	9.6	0.03
Epilepsy or mental illness	22.1	23.0	0.02	21.5	22.6	0.03	22.0	23.0	0.02
History of DVT/PE	2.5	2.5	0.00	2.3	2.7	0.02	2.4	2.5	0.00
Chronic kidney disease†	10.6	10.4	− 0.01	9.6	9.8	0.01	10.7	10.8	0.00
Asthma/Chronic obstructive pulmonary disease	15.7	15.9	0.00	15.3	15.9	0.02	15.8	16.3	0.01
History of bleeding†	8.1	8.5	0.02	7.6	8.0	0.02	8.0	8.6	0.02
Opioid-related disorders	0.1	0.0	0.00	0.1	0.0	0.00	0.1	0.0	− 0.01
Other chronic and debilitating diseases†	7.6	7.8	0.01	7.5	7.8	0.01	7.7	8.1	0.01
Frailty (proxy)	22.8	23.5	0.02	21.4	23.7	0.06	23.1	25.2	0.05
Thyroid disease	4.9	4.6	− 0.01	5.0	5.1	0.01	5.3	5.5	0.01
Obesity†	14.0	14.4	0.01	13.7	14.1	0.01	14.1	14.4	0.01
Smoking‡	12.8	13.0	0.01	12.4	12.6	0.01	12.7	12.8	0.00
**Comedications**									
Potentially hepatotoxic drugs	79.8	80.6	0.02	79.0	79.7	0.02	79.7	80.5	0.02
Antihypertensive drugs	88.5	89.0	0.01	87.3	87.7	0.01	88.4	88.8	0.01
Antiarrhythmics	65.9	66.5	0.01	66.3	66.3	0.00	64.8	65.2	0.01
Nitrovasodilator agents	7.9	8.1	0.01	7.3	7.6	0.01	7.7	7.8	0.00
Lipid-lowering agents	48.4	48.6	0.00	47.3	46.9	− 0.01	48.2	48.2	0.00
Antiplatelet drugs	53.6	54.7	0.02	52.3	53.4	0.02	53.1	54.0	0.02
Parenteral anticoagulant	20.5	20.8	0.01	18.2	19.7	0.04	19.7	19.9	0.01
NSAIDs/Antirheumatic agents	18.4	18.6	0.00	18.6	18.3	− 0.01	17.7	17.5	0.00
Oral corticosteroids	11.9	11.5	− 0.01	11.9	11.9	0.00	12.0	12.0	0.00
Opioids/other analgesics	52.2	53.1	0.02	51.4	52.4	0.02	52.4	53.5	0.02
Antiulcer agents	49.9	50.2	0.01	48.7	49.3	0.01	50.1	51.1	0.02
Hypnotics/Anxiolytics	28.8	29.3	0.01	27.9	28.6	0.02	28.4	29.2	0.02
Homeopathy	28.8	28.7	0.00	29.3	29.4	0.00	29.8	30.3	0.01
Polymedication (at index date)									
< 5 ATC classes	44.1	45.8	0.03	47.4	48.2	0.02	44.3	45.4	0,02
5–9 ATC classes	41.2	40.4	− 0.02	39.1	38.1	− 0.02	41.1	40.4	− 0,01
≥ 10 ATC classes	14.7	13.8	− 0.02	13.5	13.6	0.00	14.6	14.2	− 0,01
**Healthcare system use**									
First OAC prescriber’s specialty									
Hospital practitioner	47.5	43.6	− 0.08	45.4	42.7	− 0.05	48.1	45.7	− 0,05
General practitioner	24.7	27.4	0.06	24.1	26.9	0.06	23.8	25.8	0,05
Private cardiologist	25.5	26.7	0.03	28.2	28.1	0.00	25.7	26.1	0,01
Other private practitioners	2.3	2.3	0.00	2.4	2.4	0.00	2.4	2.3	0,00
General practitioner visits§									
0	3.2	3.0	− 0.01	3.2	3.1	− 0.01	3.1	2.9	− 0,01
1–5	32.6	31.3	− 0.03	33.8	32.5	− 0.03	33.1	32.1	− 0,02
6–11	40.1	40.5	0.01	39.9	39.9	0.00	40.1	40.2	0,00
≥ 12	24.1	25.1	0.03	23.0	24.6	0.04	23.6	24.8	0,03
Influenza vaccination$^{\P}$	55.1	56.0	0.02	53.8	54.4	0.01	55.0	55.6	0.01

*ATC* anatomical therapeutic chemical, *DVT/PE* deep vein thrombosis/pulmonary embolism, *IPTW* inverse probability of treatment weighting, *NSAIDs* non-steroidal anti-inflammatory drugs, *OAC* oral anticoagulant, *VKA* vitamin K antagonist, *SD* standard deviation, *STD* standardized difference.

*An absolute standardized difference less than 0.1 was considered to be a negligible between-group difference.

^†^Comorbidities defined by using diagnosis ICD-10 codes from hospital discharge and specific reimbursement status data onlsy.

^‡^Smoking or alcoholism data: measured using proxies, see Supplementary Table 2.

^§^Frequency of general practitioner visits was determined during the year before the index date.

$^{\P}$During the influenza vaccination campaign directly preceding the index date.

### Data analysis

All analyses were performed for both the main and the ancillary outcome.

#### Intention-to-treat analysis

Patients were followed for up to 360 days from the day after the index date until the outcome considered, death from any cause, or 31 December 2016, whichever came first.

For each type of NOAC, a Cox proportional hazards model was used to estimate the hazard ratio (HR) and its 95% confidence interval (95%CI) for the outcome considered with VKA as the reference group. To control for confounding by baseline covariates, HRs were adjusted using inverse probability of treatment weighting (IPTW). Weights were calculated for each analysis and based on the propensity score (PS), which was estimated by a logistic regression model including all baseline covariates and predicting the probability of receiving the type of NOAC considered.

The balance in baseline covariates was compared using standardized differences, before and after weighting^32^.

Crude and IPTW-adjusted non-parametric cumulative incidence curves were estimated (i.e. 1 minus Kaplan–Meier estimator of the survival curve) and plotted and their 95%CI were determined by bootstrap analysis with 200 replications.

To check for possible variations, the association between NOAC use and the outcome considered was assessed in pre-defined subgroups, according to sex, age (patients aged 80 years or over), type of NOAC dose (standard versus reduced) at initiation, risk factor for liver disease (obesity or baseline comedication with a potentially hepatotoxic drug), as well as in patients at increased risk of bleeding (i.e. with an HAS-BLED score ≥ 3, adapted to claims data) and in frail patients.

#### Sensitivity analyses

Several sensitivity analyses were performed for the main study population. First, the main intention-to-treat analysis was further adjusted for history of alcoholism (see definition in Supplementary Table 2) in the year prior OAC initiation. Second, sensitivity with respect to the handling of extreme weights was explored by modifying initial weight truncation^33^ and by using asymmetric PS trimming^34^ (All details in Supplementary Table 4).

Third, we restricted the outcome definition (1) by excluding the R17 ICD-10 code from the primary list of the codes searched (2) by restricting the definition to acute or subacute toxic ALI (i.e. K71.0, K71.1, K71.2, K72.0) or liver transplantation only.

Finally, a per-protocol analysis was performed to try to avoid bias due to potential differential adherence to the drugs compared: follow-up was additionally censored at the time of switch between the four OAC therapies (OAC dose variations were not considered) and at treatment discontinuation (identified by a 60-day gap with no medication coverage, with the additional criterion of no reimbursement for INR monitoring during this gap for VKA patients).

All calculations were performed using SAS, version 9.4 software (SAS Institute Inc.). *P* < 0.05 (two-tailed) was considered to be statistically significant.

### Ethics approval

This observational study based on the French healthcare databases was approved by the French Data Protection Agency (*Commission Nationale de l'Informatique et des Libertés*, Cnil) and did not require patient consent or ethics committee approval. Patients and or public were not involved.

## Results

### Patient selection and characteristics

**Figure 1:**
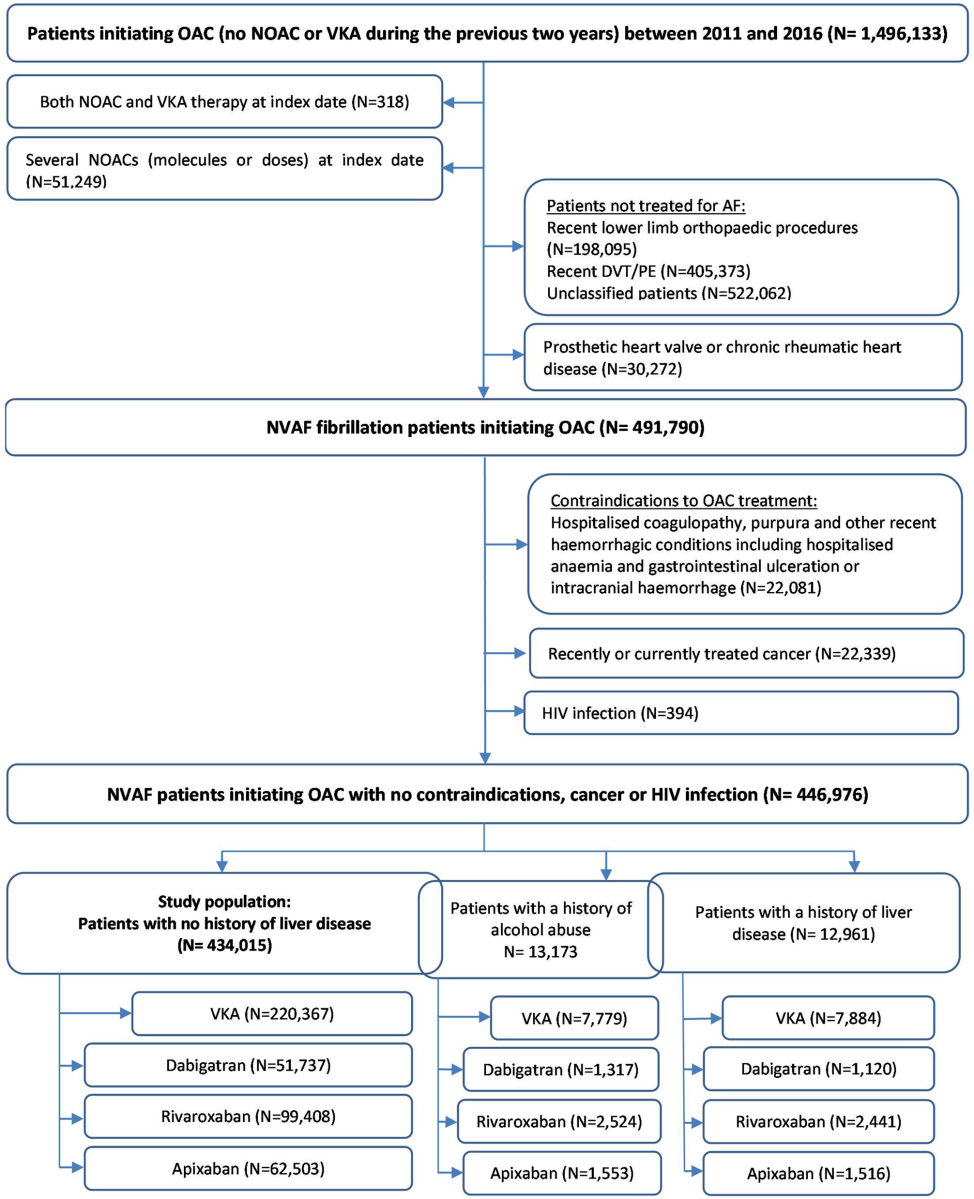
Patient selection flow chart. *DVT/PE* deep vein thrombosis/pulmonary embolism; *HIV* human immunodeficiency virus; *NVAF* non-valvular atrial fibrillation, *OAC* oral anticoagulant, *NOAC* Non-vitamin K antagonist oral anticoagulant; *VKA* vitamin K antagonist.

The main study population comprised 434,015 OAC new users with NVAF and no contraindication to OAC therapy and no prior liver disease, corresponding to 220,367 VKA, 51,737 dabigatran, 99,408 rivaroxaban and 62,503 apixaban new users. The main reasons for ineligibility were other indications or uncertain identification of the indication for OAC therapy (Fig. 1). Before weighting, in the main study population, i.e. in patients with no history of liver disease, NOAC new users were younger and had fewer comorbidities than VKA new users (See Supplementary Table 5). Across all variables included in the PS, the standardized differences ranged from − 0.54 to + 0.40 for the dabigatran/VKA cohort, − 0.55 to + 0.46 for the rivaroxaban/VKA cohort and − 0.59 to + 0.37 for the apixaban/VKA cohort. Recent history of chronic alcoholism was identified for 2.6% of VKA new users and 2.0% of each cohort of NOAC new users with no history of liver disease. During the 1-year follow-up period, discontinuing OAC treatment (switching between OAC therapies) occurred in 21.6% (8.4%), 26.5% (18.7%), 26.2% (12.6%) and 20.5% (7.8%) of VKA, dabigatran, rivaroxaban and apixaban new users, respectively.

The two additional study populations, chosen among the OAC new users with NVAF, comprised 12,961 patients with a history of liver disease and 13,173 patients with a history of chronic alcoholism, respectively (Fig. 1). Supplementary Tables 6 and 7 display the baseline characteristics of these populations.

### Main study population: OAC new users with NVAF and no prior liver disease

Table 1 displays the baseline characteristics of each OAC group after weighting; all standardized differences ranged from − 0.08 to + 0.06, indicating a good balance between treatment groups (see also Supplementary Table 4).

#### Association between OAC initiation and hospitalised ALI (main outcome)

During follow-up, a total of 218 of the 434,015 OAC new users were hospitalised for ALI (see details in Supplementary Table 8), including one case of liver transplantation in each of the VKA and dabigatran groups, corresponding to crude and IPTW-adjusted cumulative 1-year incidences reported in Table 2.

**Table 2:** Cumulative 1-year incidences of acute liver injury (and ancillary outcome) and hazard ratios for each type of NOAC compared to VKA, in patients with no prior liver disease (main study population).

Type of OAC	N patients	N events	Follow-up in days (mean ± SD)	Crude cumulative 1-year incidence with 95% CI (per 10,000)	Cumulative 1-year incidence with 95% CI after IPTW* (per 10,000)	Crude HR with 95% CI	HR after IPTW with 95% CI
**Hospitalised acute liver injury**							
VKA	220,367	117	339 ± 70	5.6 (4.7–6.7)	Dabigatran: 5.3 (4.6–6.0) Rivaroxaban: 5.1 (4.4–5.8) Apixaban: 5.3 (4.6–6.1)	Reference	Reference
Dabigatran	51,737	26	350 ± 49	5.1 (3.5–7.5)	6.2 (4.0–8.3)	0.92 (0.60–1.41)	1.17 (0.79–1.75)
Rivaroxaban	99,408	46	351 ± 48	4.7 (3.6–6.3)	7.2 (4.5–9.8)	0.85 (0.60–1.19)	1.41 (1.05–1.91)
Apixaban	62,503	29	349 ± 51	4.8 (3.3–6.9)	4.4 (2.4–6.3)	0.85 (0.57–1.28)	0.82 (0.53–1.25)
**Elevation of transaminases (proxy, ancillary outcome)**							
VKA	220,367	7537	333 ± 80	358.0 (350.2–366.1)	Dabigatran: 348.1 (342.5–353.6) Rivaroxaban: 343.2 (336.9–349.5) Apixaban: 349.3 (342.9–355.7)	Reference	Reference
Dabigatran	51,737	1353	345 ± 61	267.3 (253.6–281.7)	297.2 (280.5–314.0)	0.74 (0.70–0.79)	0.85 (0.81–0.90)
Rivaroxaban	99,408	2877	345 ± 61	295.6 (285.1–306.4)	334.1 (320.9–347.2)	0.82 (0.79–0.86)	0.97 (0.93–1.01)
Apixaban	62,503	1958	343 ± 64	320.7 (307.0–335.0)	351.7 (334.8–368.7)	0.89 (0.85–0.94)	1.01 (0.96–1.06)

*CI* confidence interval, *HR* hazard ratio, *IPTW* inverse probability of treatment weighting, *NOAC* non-vitamin K antagonist oral anticoagulant, *OAC* oral anticoagulant, *SD* standard deviation, *VKA* vitamin K antagonist.

*Calculated for each of the three comparisons.

**Figure 2:**
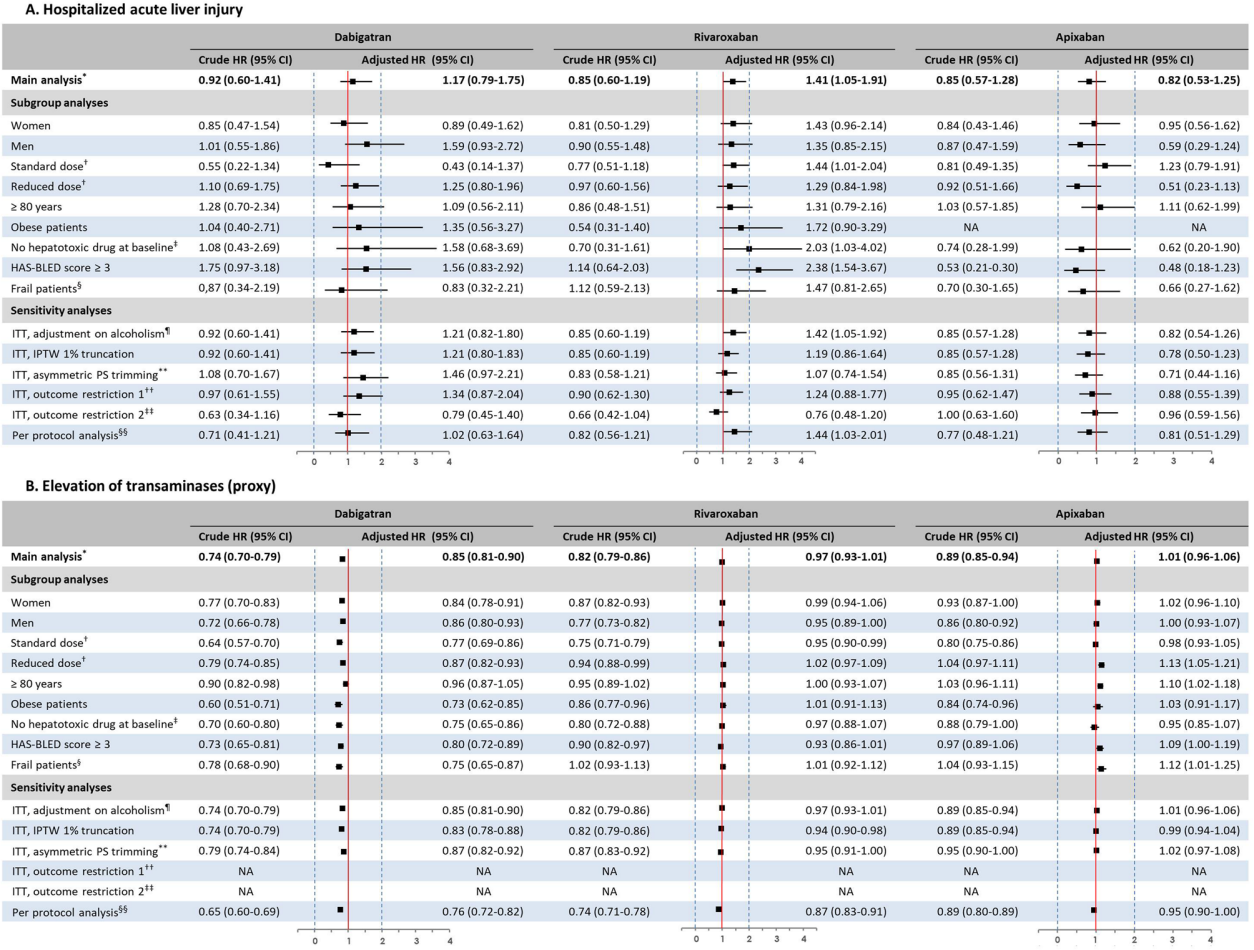
Subgroup and sensitivity analyses: Hazard ratios of acute liver injury for each type of NOAC, compared to VKA, in patients with no prior liver disease (main study population). (**A**) Hospitalized acute liver injury. (**B**) Elevation of transaminases (proxy, ancillary outcome). *Main analysis: hazard ratio adjusted by using inverse probability treatment weighting with truncation at the 0.1th and 99.9th percentiles (unlike with trimming, no patients excluded). ^†^Standard doses: dabigatran 150 mg, rivaroxaban 20 mg and apixaban 5 mg; Reduced doses: dabigatran 110 or 75 mg, rivaroxaban 15 or 10 mg and apixaban 2.5 mg. ^‡^See the list in Supplementary Table 3. ^§^See definition of frailty in Supplementary Table 2. $^{\P}$During the influenza vaccination campaign directly preceding the index date. Further adjustment on history of chronic alcoholism, see definition in Supplementary Table 2. **Asymmetric PS trimming: compared to the main analysis, 82.5% (N = 224,426), 82.7% (N = 264,550) and 81.3% (230,054) patients were included in the comparisons of dabigatran, rivaroxaban and apixaban new users versus VKA patients, respectively. ^††^Restriction to acute or unspecified toxic liver disease codes (K71.0, K71.1, K71.2, K71.6, K71.8, K71.9, K72.0, K72.9, K75.2 or K75.9) or liver transplantation. ^‡‡^Restriction to acute toxic liver disease codes (K71.0, K71.1, K71.2 or K72.0) or liver transplantation. ^§§^Follow-up censored at treatment discontinuation and switch to another oral anticoagulant. *CI* confidence interval, *ITT* intention-to-treat analysis, *PS* propensity score.

Compared with the use of VKAs, no increased risk of hospitalised ALI was observed for new users of dabigatran (adjusted HR, aHR: 1.17; 95%CI [0.79–1.75]) and apixaban (0.82 [0.53–1.25]), while an increased risk was observed in rivaroxaban new users (1.41 [1.05–1.91]) (Table 2, Fig. 2; curves in Supplementary Figure 2).

Results of subgroup analyses (Fig. 2; Supplementary Table 9) were comparable to those of the entire cohort for the dabigatran and apixaban cohorts. For rivaroxaban patients, results were statistically significant only in patients initiating NOAC standard dose, those with no hepatotoxic drug at initiation and those at increased risk of bleeding (i.e. with an HAS-BLED score ≥ 3, adapted to claims data ).

#### Association between OAC initiation and elevation of transaminases (ancillary outcome)

No association was observed between rivaroxaban (aHR: 0.97; 95%CI [0.93–1.01]) or apixaban (1.01 [0.96–1.06]) initiation and elevation of transaminases (proxy) compared to initiation of VKAs. Initiating dabigatran versus VKA therapy was associated with a reduced risk of elevation of transaminases (0.85 [0.81–0.90]). Similar results were observed in all subgroup analyses (Table 2; Supplementary Table 9).

The reduced risk and the non-significant trend towards reduced risk observed for dabigatran and rivaroxaban new users, respectively, contrasted with the increased risk (non-significant and significant, respectively) previously observed for hospitalised ALI. For apixaban analyses, the non-significant trend toward a reduced risk of hospitalised ALI differed from the null risk (or slightly increased risk in some subgroups), observed for elevation of transaminases.

#### Sensitivity analyses

Results of sensitivity analyses are reported in Fig. 2 and Supplementary Table 9.

Consistent results regarding the risk of hospitalised ALI were observed for the comparisons between dabigatran and apixaban versus VKA new users. All but one of the estimates of the association with hospitalised ALI were non-significant for the comparison between rivaroxaban and VKA cohorts. A trend towards an inverse, protective association was observed for the dabigatran and rivaroxaban cohorts when restricting the outcome definition to toxic ALI or liver transplantation only.

The results for elevation of transaminases were very similar to the estimates of the main analyses for all types of OAC comparisons.

### Additional study populations: OAC new users with NVAF and prior liver disease or with a history of chronic alcoholism

Table 3 presents the results for each population. Overall, only a few cases of hospitalised ALI were observed and incidence of hospitalised ALI was higher in both groups compared to the incidences observed in the cohort of patients with no prior liver disease. Protective associations or trends towards a protective association were observed for the risk of hospitalised ALI in both cohorts for rivaroxaban and apixaban, versus VKA. In patients with a history of chronic alcoholism, only initiating dabigatran versus VKA therapy was associated with an increased risk of hospitalised ALI (aHR: 2.88; 95%CI [1.74–4.76]), with a significantly lower risk of elevation of transaminases (0.53 [0.38–0.75]).

**Table 3:** Additional study populations: cumulative 1-year incidences of acute liver injury and hazard ratios for each type of NOAC compared to VKA, in patients with prior liver disease and in those with a history of chronic alcoholism.

	N patients	N events	Follow-up in days (mean ± SD)	Crude cumulative 1-year incidence with 95% CI (per 10,000)	Cumulative 1-year incidence with 95% CI after IPTW* (per 10,000)	Crude HR with 95% CI	HR after IPTW with 95% CI
Patients with prior liver disease (N = 12,961)							
Hospitalised acute liver injury							
VKA	7884	63	328 ± 84	86.2 (67.4–110.2)	Dabigatran: 85.3 (68.3–102.3) Rivaroxaban: 85.4 (68.7–102.0) Apixaban: 84.0 (67.6–100.3)	Reference	Reference
Dabigatran	1120	7	342 ± 66	65.3 (31.2–136.6)	109.9 (11.7–208.0)	0.76 (0.35–1.65)	1.33 (0.72–2.45)
Rivaroxaban	2441	8	342 ± 65	33.9 (17.0–67.7)	41.1 (14.0–68.3)	0.40 (0.19–0.83)	0.49 (0.25–0.96)
Apixaban	1516	4	343 ± 64	27.5 (10.3–73.1)	28.4 (4.6–52.1)	0.32 (0.12–0.87)	0.34 (0.12–0.93)
Elevation of transaminases (proxy, ancillary outcome)							
VKA	7884	21	314 ± 100	839.7 (778.6–905.4)	Dabigatran: 836.3 (787.3–885.3) Rivaroxaban: 827.9 (779.3–876.8) Apixaban: 834.6 (785.5–883.7)	Reference	Reference
Dabigatran	1120	67	330 ± 84	620.3 (491.4–781.5)	615.2 (437.6–792.7)	0.73 (0.57–0.94)	0.72 (0.56–0.93)
Rivaroxaban	2441	153	329 ± 86	647.2 (555.0–754.1)	615.6 (533.2–709.0)	0.77 (0.64–0.92)	0.74 (0.62–0.89)
Apixaban	1516	97	329 ± 84	661.9 (545.7–801.8)	678.6 (531.5–825.7)	0.78 (0.63–0.97)	0.80 (0.65–1.00)
Patients with a history of chronic alcoholism (N = 13,173)							
Hospitalised acute liver injury							
VKA	7779	48	334 ± 77	65.5 (49.4–86.8)	Dabigatran: 62.1 (49.0–75.3) Rivaroxaban: 59.6 (46.5–72.7) Apixaban: 61.8 (49.2–74.4)	Reference	Reference
Dabigatran	1317	2	342 ± 65	95.2 (54.1–167.0)	174.2 (51.8–296.6)	1.45 (0.77–2.73)	2.86 (1.73–4.75)
Rivaroxaban	2524	5	344 ± 62	20.4 (8.5–48.9)	35.9 (4.5–67.4)	0.31 (0.13–0.79)	0.61 (0.29–1.26)
Apixaban	1553	3	347 ± 55	19.7 (6.4–61.0)	8.6 (0.7–16.5)	0.30 (0.09–0.97)	0.14 (0.03–0.80)
Elevation of transaminases (proxy, ancillary outcome)							
VKA	7779	402	325 ± 88	546.6 (496.9–601.1)	Dabigatran: 534.5 (493.2–575.6) Rivaroxaban: 527.9 (488.2–567.7) Apixaban : 536.0 (495.4–576.6)	Reference	Reference
Dabigatran	1317	37	339 ± 69	295.8 (215.2–406.1)	299.1 (153.8–444.4)	0.52 (0.37–0.73)	0.54 (0.38–0.75)
Rivaroxaban	2524	111	336 ± 75	456.8 (380.8–547.7)	565.4 (448.1–682.7)	0.83 (0.67–1.02)	1.07 (0.88–1.30)
Apixaban	1553	80	338 ± 70	531.6 (429.2–657.5)	569.5 (436.4–702.5)	0.96 (0.76–1.23)	1.06 (0.84–1.34)

*Calculated for each of the three comparisons.

*CI* confidence interval, *HR* hazard ratio, *IPTW* inverse probability of treatment weighting, *NOAC* non-vitamin K antagonist oral anticoagulant, *SD* standard deviation, *VKA* vitamin K antagonist.

## Discussion

### Main findings

In this large cohort study based on health data for nearly 450,000 OAC new users with NVAF, hospitalised ALI was rare and the incidence of ALI was much higher in patients with a history of liver disease or alcoholism than in those with no history of liver disease.

No increased risk of hospitalised ALI was observed among NOAC patients during the first year after OAC initiation compared to those initiating VKA therapy, except in patients with no history of liver disease initiating rivaroxaban (association no longer significant in sensitivity analyses) and those with a history of chronic alcoholism initiating dabigatran.

### Comparison with post-marketing literature

Unlike studies based on pharmacovigilance data that found an increased risk of ALI for NOACs, especially for rivaroxaban^7,8^, the two studies based on administrative data published so far, found no increased risk of liver injury with NOAC versus VKA^16^. Overall, the results of this study that focused exclusively on ALI, are reassuring and consistent with those of these two observational studies and with clinical trial data^35^. However, in the main analysis, an increased risk was observed in rivaroxaban patients with no prior liver disease compared to VKA patients. This positive association was no longer observed when using trimming or modified weight truncation, suggesting that the risk difference in the main analysis was mainly due to a very small number of patients treated contrary to the prediction.

### Comparison between results of the main and ancillary outcomes

With the ancillary outcome, a proxy for elevation of transaminases, a protective association for dabigatran and a trend towards a protective association for rivaroxaban were observed in the main population, but no association was observed for apixaban. These results could actually reflect the differential frequency of contact with the healthcare system between NOAC and VKA therapy (no regular blood monitoring needed for NOAC patients in contrast with VKA patients), but also between the various NOAC (channelling of dabigatran and rivaroxaban towards less severe patients compared to apixaban, or when compared to VKA therapy). Closer clinical follow-up of VKA patients may have led to earlier detection and monitoring of some cases of ALI, which may have prevented hospitalisation. It could constitute another partial explanation for the slightly increased risk of hospitalised ALI observed in the main population for rivaroxaban, together with a protective association for the ancillary outcome. This explanation could also apply to the increased risk of ALI observed in dabigatran patients with a history of chronic alcoholism, among whom the strongest significant protective association was observed for the ancillary outcome, while consistent results were observed after PS trimming. Furthermore, patients who experienced the ancillary outcome in the main analysis were more than tenfold likely to be hospitalised for ALI and liver imaging (ultrasound, computed tomography or MRI scan) was performed in 37.2% of these patients during the 180-day period following the first of the three consecutive blood tests for transaminases (data not shown).

### Strengths and limitations

Although the risk of confounding by indication is limited in this study evaluating unwanted side effects of treatment, channelling bias due to preferential prescription of NOAC over VKA towards younger and less severe patients was observed. Analyses were therefore adjusted by using the IPTW method within an active comparator new user design^36^. IPTW is also known to be useful in the case of rare outcomes such as ALI^37^.

Although the PS included covariates associated with the patients’ functional and cognitive status or healthcare system consumption, residual confounding by unmeasured or incompletely captured factors, such as frailty, alcohol consumption or healthcare and sociocultural behaviours, cannot be excluded. Weight truncation and PS trimming as well as numerous restrictions were therefore used to improve comparability of patients and try to further reduce unmeasured confounding^34,38^, leading to similar conclusions. As intention-to-treat analysis may bias estimates towards the null value, outcomes were assessed during a short 1-year follow-up period and a per-protocol analysis was also performed with IPTCW adjustment.

We consider the use of the ancillary outcome to be a strength of this study, as it helped us to better interpret the results observed for hospitalised ALI. It could be useful to consider this type of tool in future comparative studies between NOAC and VKA before drawing any conclusions.

As with many other claims databases, the actual patient consumption of reimbursed drugs cannot be verified. None of the ICD-10 codes used to define ALI have been validated in the SNDS database, with the corresponding risk of misclassification. However, our outcome definition was restricted to acute liver outcomes identified exclusively by a principal hospital discharge code, which should have maximized the validity of the identified cases of ALI, especially in patients with no prior liver disease, as suggested by studies based on other databases^27,28^. The results of the sensitivity analyses using narrower ALI definitions were also in line with those of the main analyses.

This is the first observational study to assess the comparative risk of ALI in the cohort of patients with a history of chronic alcoholism. However, this cohort, as well as the cohort of patients with prior liver disease, comprised small sample sizes. The results, based on very small numbers of cases, should therefore be interpreted cautiously and in the light of the potential benefit of NOAC therapy in patients with cirrhosis^39^.

Finally, our results regarding the comparative risk of ALI in the various cohorts cannot be extrapolated beyond the first year after OAC initiation.

## Conclusion

Overall, the results of this large cohort study based on the French healthcare database do not suggest an increased risk of ALI in NOAC patients with NVAF compared to VKA patients in the first year after treatment initiation, irrespective of a history of liver disease. The risk of ALI, as a potential adverse drug reaction, may therefore not justify preferring one particular OAC therapy rather than another in these patients. Differential clinical monitoring between NOAC and VKA patients might partly explain the increased risk of ALI observed in dabigatran patients with a history of chronic alcoholism.

## Supplementary information

Supplementary information.

## Data Availability

No additional data are available directly from the authors. Permanent access to the French healthcare databases is automatically granted to certain government agencies, public institutions and public service authorities. Temporary access for studies and research is possible upon request to the national health data institute (INDS). All databases used in this study only contained anonymous patient records.
