# Supplementary material for: Oral anticoagulants and risk of acute liver injury in patients with nonvalvular atrial fibrillation: a propensity-weighted nationwide cohort study
Source: Sci Rep. 2020 Jul 15;10:11624. doi: 10.1038/s41598-020-68304-8 (PMC7363898; doi:10.1038/s41598-020-68304-8)

**Supplementary Materials**

**Oral anticoagulants and risk of acute liver injury in patients with nonvalvular atrial fibrillation: a propensity-weighted nationwide cohort study.**

Géric Maura^1^ (0000-0001-6597-5122), Marc Bardou^2,3^ (0000-0003-0028-1837), Cécile Billionnet^1^, Alain Weill MD^4^ (0000-0001-8687-9092), Jérôme Drouin^4^, Anke Neumann (0000-0002-6697-8023)^4^.

^1^ French National Health Insurance (Caisse Nationale de l’Assurance Maladie, Cnam), 75 986 Paris Cedex 20, France;

^2^ Clinical Investigation Center, Clinical Epidemiology/Clinical Trials Unit, Dijon-Bourgogne University Hospital, 21 000 Dijon, France;

^3^ Division of Gastroenterology, Dijon-Bourgogne University Hospital, 21 000 Dijon, France;

^4^ EPI-PHARE Epidemiology of Health Products, French National Agency for Medicines and Health Products Safety (ANSM) and French National Health Insurance (CNAM), 93 200 Saint-Denis, France

# Supplementary Materials

**Additional methods**

Supplementary Table 1. French national healthcare database (Système National des Données de Santé, SNDS).

Supplementary Table 2. Definitions used to identify the study population and the covariates in the French healthcare databases

Supplementary Table 3. List of considered potentially hepatotoxic drugs

Supplementary Table 4. Additional methods for the sensitivity analysis dealing with weight truncation and asymmetric propensity score trimming

Supplementary Figure 1. Study design diagram

**Additional results**

Supplementary Table 5. Baseline characteristics according to the type of OAC before and after inverse probability of treatment weighting in patients with no prior liver disease.

Supplementary Table 6. Baseline characteristics according to the type of OAC before and after inverse probability of treatment weighting in patients with prior liver diseases (first additional study population)

Supplementary Table 7. Baseline characteristics according to the type of OAC before and after inverse probability of treatment weighting in patients in patients with history of alcohol abuse (second additional study population)

Supplementary Table 8. Acute liver injury outcome: number of patients by ICD-10 diagnostic code and patients with liver transplantation.

Supplementary Table 9. Subgroup and sensitivity analyses: number of events and one-year cumulative incidences of hospitalized acute liver injury for each

Supplementary Figure 2. Cumulative incidence of hospitalized acute liver injury by type of oral anticoagulant up to one year after treatment initiation in patients with no prior liver disease (main study population)

**Additional Methods**

## Supplementary Table 1. French national healthcare database (*Système National des Données de Santé, SNDS*).

| **Database Feature** | | **The SNDS database** |
| --- | --- | --- |
| **Database population (% of population covered in the country)** | | Around 66.6 million in 2018 (100%)  In France, national health insurance (*Assurance Maladie*) provides mandatory health insurance cover for the entire population living in France; all individuals are affiliated from birth to death, irrespective of healthcare provider, age, socioeconomic status or retirement status. *Assurance Maladie* manages the French national insurance database, which, linked to the national hospital discharge summaries database and the national death registry, constitute the French healthcare databases. A 13-digit “NIR” number is used as unique healthcare identifier to identify each beneficiary throughout the individual’s lifetime. These databases therefore contain data on all health spending reimbursements for the entire population living in France. These data are also used for reimbursement purposes, with the corresponding advantages in terms of the completeness and quality control checks of timely produced data. |
| **Data used for the present study** | | Data from ‘Régime général’, around 54 million beneficiaries.  French national health insurance is divided into several specific schemes according to the beneficiaries’ occupational sector. The ‘Régime général’ scheme is the largest. |
| **Database type** | | French health insurance system database (administrative/reimbursement data) linked to the French hospital discharge database (hospital medical data). |
| **Coding systems** | | Drugs: Anatomical Therapeutic Chemical (ATC) classification  Diseases : International Classification of Diseases, 10th edition (ICD-10);  Medical procedures and imaging: French CCAM classification systems for medical procedures and imaging.  Others (laboratory tests etc.): specific French classification coding systems (e.g. NABM for lab test) |
| **Description of data** | | Individualised, anonymous, and comprehensive data on all health spending reimbursements |
|  | **Administrative data** | Date of birth, gender, and vital status; |
|  | **Data on drugs** | All reimbursed drugs dispensed in community pharmacies, encoded according to ATC classification; the packaging of each product is identified by means of a national specific pack identifier code providing information on the name of the product, active ingredient and dose in each pill, number of pills, and route of administration, but not the prescribed dose. Reimbursement for drugs dispensed during hospital stay and corresponding to a specific list of high-cost drugs (antineoplastic drugs or drugs indicated in costly long-term diseases) is also available and drugs are also coded according to ATC classification. |
|  | **Medical diagnoses** | Medical diagnosis information is available from two independent sources encoded according to the ICD-10:   1. discharge diagnoses from hospitalisation data; 2. diagnoses corresponding to specific administrative reimbursement statuses related to disability, work accidents, occupational disease, long periods of sick leave and eligibility for 100% reimbursement of severe and costly long-term diseases (list of 31 groups of diseases/diseases called “Affections longue durée, ALD”) such as, for example, AF, coronary heart disease, multiple sclerosis, epilepsy, HIV infection or cancer. |
|  | **Others** | The SNDS database also indicates medical procedures performed in the ambulatory setting or during a hospital stay, as well laboratory tests performed in the ambulatory setting, encoded according to the French classification systems. |
|  | **Data not available** | The medical indication for drug reimbursements, the results of medical procedures or laboratory tests and diagnoses from primary healthcare visits are not available. Clinical data such as BMI, patient height or previous family history are not available either. |

## Supplementary Table 2. Definitions used to identify the study population and the covariates in the French healthcare databases.

| **Covariates*** | | | **Hospital discharge diagnoses†** | **Diagnoses related to specific reimbursement status**‡ | **Specific procedures (CCAM codes) or drug reimbursements (ATC)** |
| --- | --- | --- | --- | --- | --- |
| **Definition of the study population** | | |  |  |  |
| **Non valvular atrial fibrillation** | | |  |  |  |
| Atrial fibrillation | | | I48 | I48 | Radiofrequency ablation, cardioversion |
| Lower limb orthopaedic procedures *(6-week rolling pre-index period)* | | |  |  | Lower limb orthopaedic surgery or procedures |
|  | With exclusion of: | |  |  |  |
|  |  | Deep vein thrombosis/pulmonary embolism *(6-week rolling pre-index period)* | I26, I80 (except I80.0), I81, I82 | I26, I80, I81, I82 | Lower limb venous ultrasonography, pulmonary/lower limb angiography, ventilation/perfusion scan |
|  |  | Prosthetic heart valve or chronic rheumatic heart disease | Z95.2, Z95.3, Z95.4 |  | Heart valve repair or replacement surgeries, including with bioprosthetic valve |
| **Exclusion criteria** | | |  |  |  |
| Hospitalised coagulopathy, purpura and other recent haemorrhagic conditions including hospitalised anaemia and gastrointestinal ulceration or intracranial haemorrhage *(6-week pre-index period)* | | | D50, D51, D52, D53, D55, D56, D57, D58, D59, D60, D62, D63, D64, D65 to D69, K25, K26, K27, K28; K29.0, I60, I61, I62, S06.4, S06.5, S06.6. |  |  |
| HIV infection | | | B20, B21, B22, B23, B24 | B20, B21, B22, B23, B24 | All antiviral agents for systemic use against HIV including PIs, NNRTIs, NnRTIs and other antivirals against HIV |
| Recently or currently treated cancer *(2-year rolling pre-index period)* | | | C00-D09, D37-D48, Z510, Z511 | C00-D09, D37-D48 | Radiotherapy procedures |
| History of liver disease (main population only) | | |  |  |  |
|  | Diseases of liver | | K70 to K 77, Z94.4 | K70 to K 77, Z94.4 |  |
|  | Viral hepatitis | | B15, B16, B17, B18, B19 | B15, B16, B17, B18, B19 | Antiviral agents for systemic use against hepatitis B virus or chronic hepatitis C virus (ribavirin, [peg]interferons alpha, NnRTIs and DAA) |
|  | Disorders of copper or iron metabolism | | E83.0, E83.1 | E83.0, E83.1 | CCAM codes for venipuncture: « FEJF003 » or« FEJF006 » |
| **History of liver disease : patients retained for the first additional population** | | | |  |  |
|  | | | K70 to K 77, B15, B16, B17, B18, B19, Z94.4, E83.0, E83.1 | K70 to K77, B15, B16, B17, B18, B19, Z94.4, E83.0, E83.1 | Antiviral agents for systemic use against hepatitis B virus or chronic hepatitis C virus (ribavirin, [peg]interferons alpha, NnRTIs and DAA)  CCAM codes« FEJF003 » or« FEJF006 » |
| **History of alcohol abuse or chronic alcoholism: proxies : patients retained for the second additional population** | | | | |  |
| 1-year pre-index rolling period: | | | F10, E244, G312, G621, G721, I426, K292, K70, K860, T51, Z502, Z714 | F10, E244, G312, G621, G721, I426, K292, K70, K860, T51, Z502, Z714 | Disulfiram, acamprosate, nalmefene and products with naltrexone licensed for adult patients with alcohol dependence |
| **Covariates (included in propensity score)** | | |  |  |  |
| **Comorbidities** | | |  |  |  |
| Ischemic heart disease (including myocardial infarction) | | | I20 to I25 | I20 to I25 | Nitrovasodilator agents: glyceryl trinitrate, pentaerithrityl tetranitrate, isosorbide dinitrate, Isosorbide mononitrate |
| Peripheral vascular disease | | | I70 to I73, E10.5, E11.5, E12.5, E13.5, E14.5 | I70 to I73 |  |
| Heart failure | | | I50 or I11.0, I13.0, I13.2, I13.9, K76.1, J81 related to I50 | I50 | Specific medications approved for heart failure including beta-blockers (bisoprolol, carvedilol, metoprolol), eplerenone, bumetanide, furosemide when licensed for heart failure only |
| Arrythmias (other than AF) | | | I44, I45, I47, I49 | I44, I45, I47, I49 |  |
|  | | |  |  |  |

**Supplementary Table 2. Definitions used to identify the study population and the covariates in the French healthcare databases (continued)**

| **Covariates*** | **Hospital discharge diagnoses†** | **Diagnoses related to specific reimbursement status**‡ | **Specific procedures (CCAM codes) or drug reimbursements (ATC)** |
| --- | --- | --- | --- |
| **Covariates (included in propensity score), continued** |  |  |  |
| **Comorbidities (continued)** |  |  |  |
| Diabetes | E10-E14 | E10-E14 | All insulins and oral and parenteral blood glucose lowering drugs *(at least 3 reimbursements during the year prior index date)* |
| ATE (ischemic stroke, arterial systemic embolism or transient ischemic attack) | I63 (except I63.6), G46 related to I63 or I69.3; I74, G45 (except G45.4) | I63, I74, G45 |  |
| Dementia or Parkinson’s disease | F00 to F03, G30, G31.1, G20 | F00 to F03, G30, G31.1, G20 | Anticholinesterases or NMDA receptor antagonists; anticholinergic or dopaminergic agents licensed for Parkinson’s disease |
| Mental illness and epilepsy | F20 to 29, F30 to 39, G40, G41 | F20-29, F30-39, G40, G41 | All antidepressants, antipsychotics, conventional mood stabilizers and antiepileptic drugs |
| DVT/PE *(beyond the 6-week rolling pre-index period used to define exclusion criteria)* | I26, I80 (except I80.0), I81, I82 | I26, I80, I81, I82 | Lower limb venous ultrasonography, pulmonary/lower limb angiography, ventilation/perfusion scan |
| Chronic kidney disease | N18, I12, I13.1, I13.2, E10.2, E11.2, E13.2, E14.2 | N18, I12 |  |
| Asthma/COPD | J44 to J46; J96 | J44 to J46; J96 | Drugs for obstructive airway diseases, inhalants: glucocorticoids, antiallergic agents, selective beta-2-adrenoreceptor agonists, xanthines, leukotriene receptor antagonists, omalizumab *(at least 3 reimbursements during the year prior index date)* |
| History of bleeding | I60 to I62, S06.3 to S06.6, I85, K25 to K29, K62.5, K66.1, K92.0 to K92.2, D62, D69.9, N02, R31, R58, H11.3, H35.6, H43.1, H45.0, H92.2, J94.2, M25.0, N92, N93.8, N93.9, N95.0, R04(.0,.1,.2,.8,.9), I32.2 |  |  |
| Opioid-related disorders | F11 | F11 |  |
| Other chronic and debilitating diseases | G35, M05-M07, M15, M45-M49, M30-M36, K50-K51, M60-M63, G40-G41 | G35, M05-M07, M15, M45-M49, M30-M36, K50-K51, M60-M63, G40-G41 |  |
| Frailty: proxies | E43, E44.0, G81-G83, L89, M80, R15, R32, R26.0 to .3, R29.6 |  | Home hospital bed, wheelchair, high level of nursing home stay, oxygen therapy, pressure ulcers, enteral nutrition |
| Thyroid disease | E00, E01, E02, E03, E04, E05, E06 | E00, E01, E02, E03, E04, E05, E06 |  |
| Obesity | E66 | E66 |  |
| Smoking (current or past consumption): proxies | F17, Z71.6, Z72.0; T65.2, J43-J44 | J43-J44 | Nicotine replacement therapies |
| History of alcoholism (sensitivity analysis only) | See ‘History of alcohol abuse’ above |  |  |
| **Comedications** |  |  |  |
| Potentially hepatotoxic drugs *(6-week pre-index period)* | See Supplementary Table 2 |  |  |
| Antihypertensive drugs *(at least 3 reimbursements during the year prior index date)* |  |  | Diuretics, beta blockers, calcium channel blockers, agents acting on the renin-angiotensin system and other antiadrenergic agents |
| Antiarrhythmics |  |  | Antiarrhythmics: class I and III, verapamil, digitalis glycosides |
| Nitrovasodilator agents |  |  | Glyceryl trinitrate, pentaerithrityl tetranitrate, isosorbide dinitrate, isosorbide mononitrate, and cardiac glycosides |
| Lipid-lowering agents |  |  | HMG CoA reductase inhibitors, fibrates, ezetimibe |

**Supplementary Table 2. Definitions used to identify the study population and the covariates in the French healthcare databases (continued)**

| **Covariates*** | **Hospital discharge diagnoses†** | **Diagnoses related to specific reimbursement status**‡ | **Specific procedures (CCAM codes) or drug reimbursements (ATC)** |
| --- | --- | --- | --- |
| **Covariates (included in propensity score), continued** |  |  |  |
| **Comedications, continued** |  |  |  |
| Antiplatelet drugs |  |  | Platelet aggregation inhibitors excl. heparin and including low-dose acetylsalicylic acid |
| Parenteral anticoagulants |  |  | Heparin agents, fondaparinux |
| NSAIDs/Antirheumatic agents |  |  | Antiinflammatory and antirheumatic products, non-steroids |
| Oral corticosteroids |  |  | Mineralo- and gluco-corticoids for systemic use |
| Opioids and other analgesics (at least 3 reimbursements during the year prior index date) |  |  | All opioid agents (including natural opium alkaloids, tramadol) and other analgesics and antipyretics (including paracetamol, salicylic acid and derivatives) |
| Antiulcer agents |  |  | Antacids and drugs for peptic ulcer and gastro-oesophageal reflux disease (including proton pump inhibitors and H2-receptor antagonists) |
| Hypnotic/Anxiolytics *(at least 3 reimbursements during the year prior index date)* |  |  | Anxiolytics, hypnotics and sedatives drugs (mainly benzodiazepines and benzodiazepine-related drugs) |
| Homeopathy |  |  | Reimbursed homeopathic treatments |
| Influenza vaccination *(during the first ‘flu vaccination campaign preceding the index date)* |  |  | All Influenza vaccines (excl. influenza, live attenuated) |
| **HAS-BLED score (descriptive purpose)** |  |  |  |
| H (hypertension) |  |  | Diuretics, beta blockers, calcium channel blockers, agents acting on the renin-angiotensin system and other antiadrenergic agents |
| A (abnormal liver/renal function) | N18 et I12 and K70, K73, K74, B18, C22 | N18 et I12 and K70, K73, K74, B18, C22 | Concomitant antiviral drugs against hepatitis viruses as defined above |
| S (stroke) | I63 (except I63.6), G46 related to I63 or I69.3; I74, G45 (except G45.4) | I63, I74, G45 |  |
| B (Prior major bleeding or predisposition) | I60-I62, S06.3-S06.6, I85, K25-K29, K62.5, K92, D62, N02, R31, R58, H11.3, H35.6, H43.1, H45.0, H92.2, J94.2, K66.1, M25.0, N92, N93.8, N93.9, N95.0, R04.0 or recent anaemia: D50, D51, D52, D53, D55, D56, D57, D58, D59, D60, D62, D63, D64 |  |  |
| L (labile INR) | NA | NA | NA |
| E (elderly, age >65 years old) |  |  |  |
| D (Drugs, alcohol concomitantly, *at index date*) | F10, E244, G312, G621, G721, I426, K292, K70, K860, T51, Z502, Z714 | F10, E244, G312, G621, G721, I426, K292, K70, K860, T51, Z502, Z714 | Disulfiram, acamprosate, nalmefene and products with naltrexone licensed for adult patients with alcohol dependence; concomitant ineracting drugs considered: NSAIDs, platelet aggregation inhibitors (including low-dose acetylsalicylic acid ) and parenteral anticoagulants |
| **History of alcohol use or chronic alcoholism (proxy, for further adjustment in sensitivity analyses)** | |  |  |
| See definition of “History of alcohol abuse or chronic alcoholism” | | | |

ATE: arterial thromboembolic events (mainly stroke); NMDA: N-methyl-D-aspartate; HIV: human immunodeficiency virus; PI: Protease inhibitor; NNRTIs: Non-nucleoside reverse transcriptase inhibitors; NnRTI: Nucleoside and nucleotide reverse transcriptase inhibitors; DAA: direct-acting antiviral; HMG CoA: 3-hydroxy-3-methyl-glutaryl-CoA; NSAIDs: Non-steroidal anti-inflammatory drugs; HCV: hepatitis C virus; HCB: hepatitis B virus

* Comorbidities were identified using a pre-index 5-year rolling period of data history (having at least one reimbursement related to the ICD-10 code considered) and concomitant medications were identified as those reimbursed at least once during the 4-month period preceding the index date, unless otherwise specified *in italics* in the Table, for clinical and/or sensitivity reasons.

† ICD-10 codes from hospital discharges diagnoses (all positions) contained in the national hospital database from medical, surgical and obstetrics and care (*PMSI champ médicine, chirurgie obstétrique*, *MCO*) or follow-up care and rehabilitation (*PMSI SSR*).

‡ ICD-10 codes from diagnoses mainly related to eligibility for 100% reimbursement of severe and costly long-term diseases (French “Affection longue durée”, ALD) and also to long periods of sick leave, disability, work accidents or occupational disease.

## Supplementary Table 3. List of considered potentially hepatotoxic drug

Rationale for making the list:

- Drugs from the category A (“*>50 case reports of liver injury associated with the use of these drugs, 81% of the drugs had >100 cases reported. Interestingly, overall, 92% of these drugs had documented positive rechallenge […] 98% had at least one convincing case that was associated with fatal outcome”*) of ‘Björnsson ES. Hepatotoxicity by Drugs: The Most Common Implicated Agents. Int J Mol Sci. 2016;17(2):224’;
- Additionally drugs from the list of ‘Biour M et al, Drug-induced liver injury; fourteenth updated edition of the bibliographic database of liver injuries and related drugs. Gastroenterol Clin Biol 2004 ;28(8-9):720-59.’ (if N≥80);
- Are not included: drugs to treat HIV or cancer as patients with HIV infection or cancer at baseline were excluded from this cohort study as well as floxuridine, flucloxacillin, chlorzoxazone, anabolic steroids and some antimicrobial sulfonamides (not available in France ) and halothane, specific antirheumatic agents gold preparation (inhospital drugs not available in the French databases).

| **Drug** | **ATC code** |
| --- | --- |
| Paracetamol (acetaminophen) alone or in association with | N02BE01, N02AA59, N02AC54, N02AX52, N02BA51, N02BE05, N02BE51, N02BE71 |
| Allopurinol seul et en association | M04AA01, M04AA51 |
| Amiodarone, dronedarone | C01BD01, C01BD07 |
| Amoxicillin-clavulanate | J01CR02 |
| Atorvastatin | C10AA05 |
| Azathioprine | L04AX01 |
| Aztreonam | J01DF01 |
| Carbamazepine | N03AF01 |
| Ceftriaxone | J01DD04 |
| Chlorpromazine | N05AA01 |
| Ciprofloxacine systémique | J01MA02 |
| Contraceptifs oraux estroprogestatifs | Starting with G03AA- or G03AB- |
| Dantrolene | M03CA01 |
| Diclofenac | M01AB05, M01AB55 |
| Disulfiram | N07BB01 |
| Erythromycine systémique, seule et en association | J01FA01, J01RA02 |
| Fluconazole | J02AC01 |
| Hydralazine | C07BA02 |
| Ibuprofen | M01AE01, N02AA59 |

**Supplementary Table 3. List of potentially hepatotoxic drug considered as baseline comedication (continued)**

| **Drug** | **ATC code** |
| --- | --- |
| Imipenem+cilastatine | J01DH51 |
| Infliximab | L04AB02 |
| Interferon alpha/Peginterferon, Interferon beta | L03AB04, L03AB05, L03AB07, L03AB08, L03AB09, L03AB10, L03AB11, L03AB13 |
| Isoniazid | J04AC01, J04AM02, J04AM03, J04AM05 |
| Ketoconazole (systemic) | J02AB02 |
| Meropenem* | J01DH02 |
| Methotrexate | L04AX03, L01BA01 |
| Methyldopa | C02AB02 |
| Minocycline | J01AA08 |
| Nimesulide | M01AX17 |
| Nitrofurantoin | J01XE01 |
| Phenytoin | N03AB02 |
| Propylthiouracil | H03BA02 |
| Quinidine | ‘P01BC’ et ‘C01BA’ with no extension, P01BC01, C01BA01 |
| Pyrazinamide | J04AM05, J04AK01 |
| Rifampin | J04AM02, J04AM05, J04AB02 |
| Simvastatin | C10AA01, C10BA02 |
| Sulfamethoxazole/Trimethoprim = cotrimoxazole | J01EE01 |
| Sulfasalazine | A07EC01 |
| Sulindac | M01AB02 |
| Telithromycin | J01FA15 |
| Ticlopidine | B01AC05 |
| Valproate | N03AG01 |

## Supplementary Table 4. Additional methods including the sensitivity analysis dealing with weight truncation and asymmetric propensity score trimming

1. **Check of the assumptions underlying inverse probability of treatment weighting (IPTW) methods:**

In this study, the hazard ratios were adjusted using the method of inverse probability of treatment weighting, which relies on four assumptions: consistency, exchangeability, positivity, and no misspecification of the model used to estimate weights (Cole SR & Hernán MA, Am J Epidemiol 2008).

Consistency is considered to be plausible for medical treatments for which manipulation is not difﬁcult to conceive.

Exchangeability cannot be formally tested but instead, we did a careful selection of confounders based on subject matter knowledge, leading to the inclusion of the 39 variables in the propensity score model.

Regarding positivity, stabilized weights were calculated with a mean close to one, no very extreme values (see the table below) and the choice of the points of truncation showed little impact of on the final effect estimate (see the description of the corresponding sensitivity analyses below, in part B, and their results in Supplementary Table 9.).

The good balance between treatment groups indicated by the standardized difference after weighting (see Supplementary Tables 5 to 7) as well as the results regarding the mean of the stabilized weights, are in favor of correct exposure model speciﬁcation.

| **Population** | **NOAC therapy (compared to VKA)** | **Before weight truncation** | | | | **After weight truncation** | | | |
| --- | --- | --- | --- | --- | --- | --- | --- | --- | --- |
|  |  | **Mean** | **Min** | **Max** | **SD** | **Mean** | **Min** | **Max** | **SD** |
| **Main : NVAF and no prior liver disease** | |  |  |  |  |  |  |  |  |
|  | Dabigatran | 1.00 | 0.30 | 68.1 | 0.6 | 1.00 | 0.36 | 7.8 | 0.5 |
|  | Rivaroxaban | 1.01 | 0.38 | 85.4 | 0.8 | 1.00 | 0.42 | 9.9 | 0.6 |
|  | Apixaban | 1.01 | 0.31 | 45.8 | 0.7 | 1.00 | 0.37 | 9.4 | 0.5 |
| **Additional population 1: NVAF and prior liver disease** | | |  |  |  |  |  |  |  |
|  | Dabigatran | 1.01 | 0.23 | 32.3 | 0.6 | 1.00 | 0.26 | 8.7 | 0.4 |
|  | Rivaroxaban | 1.01 | 0.30 | 27.4 | 0.7 | 1.00 | 0.33 | 9.0 | 0.5 |
|  | Apixaban | 1.00 | 0.25 | 18.3 | 0.4 | 1.00 | 0.30 | 6.0 | 0.4 |
| **Additional population 2: NVAF and history of chronic alcoholism** | | | |  |  |  |  |  |  |
|  | Dabigatran | 1.01 | 0.23 | 52.2 | 0.8 | 1.00 | 0.29 | 8.2 | 0.4 |
|  | Rivaroxaban | 1.01 | 0.32 | 21.4 | 0.6 | 1.00 | 0.36 | 7.8 | 0.5 |
|  | Apixaban | 1.00 | 0.29 | 18.7 | 0.5 | 1.00 | 0.33 | 7.3 | 0.4 |
|  |  |  |  |  |  |  |  |  |  |

SD: standard deviation

1. **Sensitivity analysis dealing with weight truncation and asymmetric propensity score trimming:**

| **Analyses** | | **Weight truncation** | **Asymmetric propensity score trimming** | **Rationale and calculations** |
| --- | --- | --- | --- | --- |
| **Main analysis** | |  |  |  |
| Stabilized weights and 0.1% truncation | | All weights with values above the 99.9th percentile and below the 0.1th percentile were considered to be equal to the 99.9th and 0.1th percentiles, respectively | No | With the IPTW method, atypical patients for whom the expected treatment, i.e. the type of NOAC or VKA considered, was not chosen, presented large weights. Weights were therefore first stabilized (and therefore varied around 1) and then truncated: all weights with values above the 99.9th percentile and below the 0.1th percentile were considered to be equal to the 99.9th and 0.1th percentiles, respectively.  Compared to the main population of the main analyses, no patients are excluded. |
| **Sensitivity analyses** | |  |  |  |
| 1 | Stabilized weights and 0.5% truncation (instead of 0.1%) | Truncation at the 0.5th and 99.5th percentiles applied | No | In sensitivity analyses, further truncation was applied to test the consistency of the results. |
| 2 | Stabilized weights and 1% truncation (instead of 1%) | Truncation at the 1st and 99th percentiles was applied | No |  |
| 3 | Asymmetric propensity score trimming and stabilized weights | No | Yes | Asymmetric propensity score trimming is a method that involves dropping the individuals with the most extreme propensity score values in the two treatment groups, excluding persons treated contrary to prediction.  Unlike with truncation, some patients are therefore excluded from the main population as defined for the main analysis. This method has been shown to reduce unmeasured confounding in certain situations and due to the nature of administrative data used in this study, was worth applying as sensitivity analysis.  Here the lower limit for removal was determined as the 2.5th percentile of the propensity score values among the patients treated by the type of NOAC considered and the upper limit by the 97.5th percentile among the patients treated by VKA. The HR was then estimated from the remaining patients, using the stabilized weights. |

## Supplementary Figure 1. Study design diagram


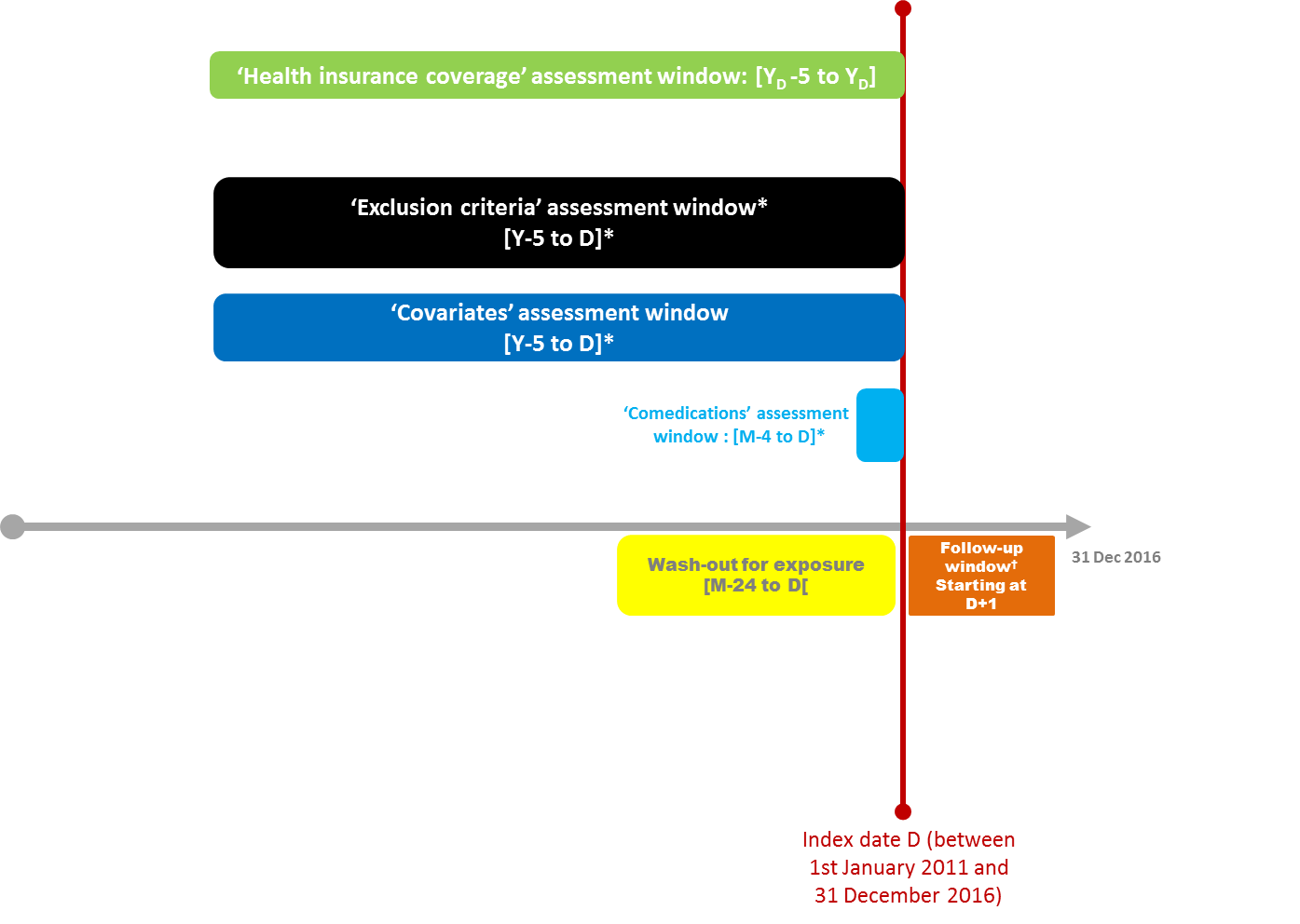


*See details in Supplementary Table 2.

†Follow-up for up to 360 days until the considered outcome, death from any cause, or 31 December 2016, whichever came first.

Y_D_= Calendar year of D; Y-1: the year prior to index date D, Y-X: the X^nth^ year prior to index date D; M-1: the month prior to index date, M-X: the x^nth^ month prior to index date D.

**Additional Results**

## Supplementary Table 5. Baseline characteristics according to the type of OAC before and after inverse probability of treatment weighting in patients with no prior liver disease.

| **Characteristics**  **(column %, unless stated otherwise)** | | **Dabigatran *versus* VKA (N=51,737 *vs* 220,367)** | | | | | | **Rivaroxaban *versus* VKA (N= 99,408 *vs* 220,367)** | | | | | | **Apixaban *versus* VKA (N= 62,503 *vs* 220,367)** | | | | | |
| --- | --- | --- | --- | --- | --- | --- | --- | --- | --- | --- | --- | --- | --- | --- | --- | --- | --- | --- | --- |
|  |  | **Before IPTW** | | | **After IPTW** | | | **Before IPTW** | | | **After IPTW** | | | **Before IPTW** | | | **After IPTW** | | |
|  |  | **VKA** | **Dab** | **STD*** | **VKA** | **Dab** | **STD*** | **VKA** | **Riv** | **STD*** | **VKA** | **Riv** | **STD*** | **VKA** | **Api** | **STD*** | **VKA** | **Api** | **STD*** |
| **Sociodemographic data** | |  |  |  |  |  |  |  |  |  |  |  |  |  |  |  |  |  |  |
| Age (years), mean  (SD) | | 76.8 (11.2) | 74.0 (11.5) | -0.25 | 76.3 (11.3) | 76.6 (11.1) | 0.03 | 76.8 (11.2) | 73.0 (11.7) | -0.33 | 75.7 (11.5) | 76.1 (11.6) | 0.04 | 76.8 (11.2) | 75.1 (11.4) | -0.15 | 76.4 (11.3) | 76.9 (11.1) | 0.04 |
|  | 18-54 | 4.2 | 6.2 | 0.09 | 4.5 | 4.0 | -0.02 | 4.2 | 7.0 | 0.13 | 5.0 | 4.6 | -0.02 | 4.2 | 5.1 | 0.04 | 4.4 | 3.8 | -0.03 |
|  | 55-64 | 10.1 | 12.8 | 0.09 | 10.6 | 10.2 | -0.01 | 10.1 | 13.8 | 0.11 | 11.2 | 10.6 | -0.02 | 10.1 | 11.4 | 0.04 | 10.4 | 9.8 | -0.02 |
|  | 65-74 | 21.0 | 26.4 | 0.13 | 22.0 | 21.7 | -0.01 | 21.0 | 28.8 | 0.18 | 23.3 | 22.5 | -0.02 | 21.0 | 26.1 | 0.12 | 22.1 | 21.5 | -0.02 |
|  | 75-79 | 16.9 | 18.1 | 0.03 | 17.1 | 16.9 | -0.01 | 16.9 | 17.6 | 0.02 | 17.1 | 16.9 | -0.01 | 16.9 | 16.8 | 0.00 | 16.8 | 16.9 | 0.00 |
|  | 80-84 | 21.1 | 19.1 | -0.05 | 20.8 | 21.2 | 0.01 | 21.1 | 17.5 | -0.09 | 20.0 | 20.3 | 0.01 | 21.1 | 19.5 | -0.04 | 20.8 | 21.0 | 0.01 |
|  | 85-89 | 17.4 | 12.5 | -0.14 | 16.5 | 17.1 | 0.02 | 17.4 | 10.8 | -0.19 | 15.4 | 16.1 | 0.02 | 17.4 | 14.3 | -0.09 | 16.7 | 17.5 | 0.02 |
|  | ≥90 | 9.4 | 4.9 | -0.17 | 8.5 | 8.9 | 0.01 | 9.4 | 4.5 | -0.19 | 7.9 | 9.0 | 0.04 | 9.4 | 6.9 | -0.09 | 8.8 | 9.5 | 0.02 |
| Female sex | | 53.6 | 51.4 | -0.04 | 53.2 | 53.7 | 0.01 | 53.6 | 49.9 | -0.07 | 52.5 | 53.5 | 0.02 | 53.6 | 52.8 | -0.02 | 53.4 | 54.0 | 0.01 |
| Deprivation index | |  |  |  |  |  |  |  |  |  |  |  |  |  |  |  |  |  |  |
|  | Quintile 1 (least deprived) | 16.2 | 17.9 | 0.05 | 16.5 | 16.5 | 0.00 | 16.2 | 18.9 | 0.07 | 17.0 | 16.9 | 0.00 | 16.2 | 17.3 | 0.03 | 16.4 | 16.3 | 0.00 |
|  | Quintile 2 | 18.6 | 18.2 | -0.01 | 18.5 | 18.6 | 0.00 | 18.6 | 20.0 | 0.04 | 19.0 | 18.9 | 0.00 | 18.6 | 19.5 | 0.02 | 18.8 | 18.6 | -0.01 |
|  | Quintile 3 | 19.8 | 19.6 | 0.00 | 19.8 | 19.4 | -0.01 | 19.8 | 19.5 | -0.01 | 19.7 | 19.4 | -0.01 | 19.8 | 20.2 | 0.01 | 19.9 | 19.6 | -0.01 |
|  | Quintile 4 | 21.3 | 20.6 | -0.02 | 21.2 | 21.2 | 0.00 | 21.3 | 19.8 | -0.04 | 20.9 | 21.0 | 0.00 | 21.3 | 20.5 | -0.02 | 21.2 | 21.4 | 0.01 |
|  | Quintile 5 (most deprived) | 22.5 | 21.8 | -0.02 | 22.4 | 22.7 | 0.01 | 22.5 | 20.6 | -0.05 | 22.0 | 22.3 | 0.01 | 22.5 | 21.5 | -0.03 | 22.3 | 22.6 | 0.01 |
|  | Overseas departments | 1.5 | 1.8 | 0.02 | 1.6 | 1.6 | 0.00 | 1.5 | 1.4 | -0.01 | 1.5 | 1.5 | 0.00 | 1.5 | 1.1 | -0.04 | 1.4 | 1.5 | 0.00 |
| **Comorbidities** | |  |  |  |  |  |  |  |  |  |  |  |  |  |  |  |  |  |  |
| Ischaemic heart disease | | 30.2 | 20.9 | -0.21 | 28.4 | 28.7 | 0.01 | 30.2 | 20.3 | -0.23 | 27.1 | 27.5 | 0.01 | 30.2 | 22.2 | -0.18 | 28.4 | 28.6 | 0.00 |
| Vascular disease | | 36.5 | 25.3 | -0.24 | 34.4 | 35.0 | 0.01 | 36.5 | 24.7 | -0.26 | 32.9 | 33.6 | 0.02 | 36.5 | 27.0 | -0.21 | 34.4 | 35.0 | 0.01 |
| Heart failure | | 38.9 | 28.4 | -0.22 | 36.9 | 36.6 | 0.00 | 38.9 | 29.3 | -0.20 | 35.8 | 36.2 | 0.01 | 38.9 | 34.4 | -0.09 | 37.8 | 38.0 | 0.00 |
| Arrhythmias (other than AF)† | | 23.4 | 19.7 | -0.09 | 22.7 | 23.0 | 0.01 | 23.4 | 18.5 | -0.12 | 21.9 | 22.4 | 0.01 | 23.4 | 19.9 | -0.08 | 22.6 | 23.3 | 0.02 |
| Diabetes | | 24.8 | 19.8 | -0.12 | 23.9 | 24.4 | 0.01 | 24.8 | 19.7 | -0.12 | 23.3 | 23.6 | 0.01 | 24.8 | 21.0 | -0.09 | 24.0 | 24.0 | 0.00 |

**Supplementary Table 5 (continued).**

| **Characteristics**  **(column %, unless stated otherwise)** | **Dabigatran *versus* VKA (N=51,737 *vs* 220,367)** | | | | | | **Rivaroxaban *versus* VKA (N= 99,408 *vs* 220,367)** | | | | | | **Apixaban *versus* VKA (N= 62,503 *vs* 220,367)** | | | | | |
| --- | --- | --- | --- | --- | --- | --- | --- | --- | --- | --- | --- | --- | --- | --- | --- | --- | --- | --- |
|  | **Before IPTW** | | | **After IPTW** | | | **Before IPTW** | | | **After IPTW** | | | **Before IPTW** | | | **After IPTW** | | |
|  | **VKA** | **Dab** | **STD*** | **VKA** | **Dab** | **STD*** | **VKA** | **Riv** | **STD*** | **VKA** | **Riv** | **STD*** | **VKA** | **Api** | **STD*** | **VKA** | **Api** | **STD*** |
| **Comorbidities (continued)** |  |  |  |  |  |  |  |  |  |  |  |  |  |  |  |  |  |  |
| History of ATE† | 15.2 | 11.8 | -0.10 | 14.6 | 15.5 | 0.03 | 15.2 | 9.1 | -0.19 | 13.4 | 14.4 | 0.03 | 15.2 | 12.5 | -0.08 | 14.6 | 15.4 | 0.02 |
| Dementia or Parkinson’s disease | 9.5 | 5.8 | -0.14 | 8.8 | 9.4 | 0.02 | 9.5 | 5.5 | -0.15 | 8.3 | 9.2 | 0.03 | 9.5 | 6.0 | -0.13 | 8.7 | 9.6 | 0.03 |
| Epilepsy or mental illness | 22.8 | 18.9 | -0.10 | 22.1 | 23.0 | 0.02 | 22.8 | 18.3 | -0.11 | 21.5 | 22.6 | 0.03 | 22.8 | 18.9 | -0.10 | 22.0 | 23.0 | 0.02 |
| History of DVT/PE | 2.8 | 1.0 | -0.13 | 2.5 | 2.5 | 0.00 | 2.8 | 1.1 | -0.13 | 2.3 | 2.7 | 0.02 | 2.8 | 0.8 | -0.15 | 2.4 | 2.5 | 0.00 |
| Chronic kidney disease† | 12.4 | 3.4 | -0.34 | 10.6 | 10.4 | -0.01 | 12.4 | 3.5 | -0.33 | 9.6 | 9.8 | 0.01 | 12.4 | 4.7 | -0.28 | 10.7 | 10.8 | 0.00 |
| Asthma/COPD | 16.7 | 11.8 | -0.14 | 15.7 | 15.9 | 0.00 | 16.7 | 12.2 | -0.13 | 15.3 | 15.9 | 0.02 | 16.7 | 12.9 | -0.11 | 15.8 | 16.3 | 0.01 |
| History of bleeding† | 8.5 | 6.1 | -0.09 | 8.1 | 8.5 | 0.02 | 8.5 | 5.5 | -0.12 | 7.6 | 8.0 | 0.02 | 8.5 | 6.0 | -0.10 | 8.0 | 8.6 | 0.02 |
| Opioid-related disorders | 0.1 | 0.0 | -0.01 | 0.1 | 0.0 | 0.00 | 0.1 | 0.0 | 0.00 | 0.1 | 0.0 | 0.00 | 0.1 | 0.0 | -0.01 | 0.1 | 0.0 | -0.01 |
| Other chronic and debilitating diseases† | 7.9 | 6.1 | -0.07 | 7.6 | 7.8 | 0.01 | 7.9 | 6.3 | -0.06 | 7.5 | 7.8 | 0.01 | 7.9 | 6.9 | -0.04 | 7.7 | 8.1 | 0.01 |
| Frailty (proxy) | 24.9 | 13.7 | -0.28 | 22.8 | 23.5 | 0.02 | 24.9 | 13.3 | -0.30 | 21.4 | 23.7 | 0.06 | 24.9 | 16.9 | -0.20 | 23.1 | 25.2 | 0.05 |
| Thyroid disease | 5.0 | 4.3 | -0.03 | 4.9 | 4.6 | -0.01 | 5.0 | 5.1 | 0.00 | 5.0 | 5.1 | 0.01 | 5.0 | 6.2 | 0.05 | 5.3 | 5.5 | 0.01 |
| Obesity† | 14.5 | 11.6 | -0.09 | 14.0 | 14.4 | 0.01 | 14.5 | 11.7 | -0.08 | 13.7 | 14.1 | 0.01 | 14.5 | 12.5 | -0.06 | 14.1 | 14.4 | 0.01 |
| Smoking‡ | 13.4 | 10.0 | -0.11 | 12.8 | 13.0 | 0.01 | 13.4 | 10.0 | -0.11 | 12.4 | 12.6 | 0.01 | 13.4 | 10.0 | -0.11 | 12.7 | 12.8 | 0.00 |
| **Comedications** |  |  |  |  |  |  |  |  |  |  |  |  |  |  |  |  |  |  |
| Potentially hepatotoxic drugs | 80.6 | 76.3 | -0.11 | 79.8 | 80.6 | 0.02 | 80.6 | 74.9 | -0.14 | 79.0 | 79.7 | 0.02 | 80.6 | 76.3 | -0.10 | 79.7 | 80.5 | 0.02 |
| Antihypertensive drugs | 89.5 | 84.4 | -0.15 | 88.5 | 89.0 | 0.01 | 89.5 | 82.1 | -0.21 | 87.3 | 87.7 | 0.01 | 89.5 | 84.6 | -0.15 | 88.4 | 88.8 | 0.01 |
| Antiarrhythmics or cardiac glycosides | 64.6 | 71.5 | 0.15 | 65.9 | 66.5 | 0.01 | 64.6 | 70.0 | 0.12 | 66.3 | 66.3 | 0.00 | 64.6 | 65.1 | 0.01 | 64.8 | 65.2 | 0.01 |
| Nitrovasodilator agents | 8.5 | 5.3 | -0.13 | 7.9 | 8.1 | 0.01 | 8.5 | 4.6 | -0.16 | 7.3 | 7.6 | 0.01 | 8.5 | 5.0 | -0.14 | 7.7 | 7.8 | 0.00 |
| Lipid-lowering agents | 49.1 | 45.4 | -0.07 | 48.4 | 48.6 | 0.00 | 49.1 | 43.0 | -0.12 | 47.3 | 46.9 | -0.01 | 49.1 | 45.0 | -0.08 | 48.2 | 48.2 | 0.00 |
| Antiplatelet drugs | 54.5 | 49.4 | -0.10 | 53.6 | 54.7 | 0.02 | 54.5 | 46.5 | -0.16 | 52.3 | 53.4 | 0.02 | 54.5 | 47.4 | -0.14 | 53.1 | 54.0 | 0.02 |
| Parenteral anticoagulant (heparin) | 24.0 | 5.6 | -0.54 | 20.5 | 20.8 | 0.01 | 24.0 | 5.2 | -0.55 | 18.2 | 19.7 | 0.04 | 24.0 | 4.2 | -0.59 | 19.7 | 19.9 | 0.01 |
| NSAIDs or antirheumatic agents | 17.7 | 21.4 | 0.09 | 18.4 | 18.6 | 0.00 | 17.7 | 20.4 | 0.07 | 18.6 | 18.3 | -0.01 | 17.7 | 17.5 | -0.01 | 17.7 | 17.5 | 0.00 |

**Supplementary Table 5 (continued).**

| **Characteristics**  **(column %, unless stated otherwise)** | | **Dabigatran *versus* VKA (N=51,737 *vs* 220,367)** | | | | | | **Rivaroxaban *versus* VKA (N= 99,408 *vs* 220,367)** | | | | | | **Apixaban *versus* VKA (N= 62,503 *vs* 220,367)** | | | | | |
| --- | --- | --- | --- | --- | --- | --- | --- | --- | --- | --- | --- | --- | --- | --- | --- | --- | --- | --- | --- |
|  |  | **Before IPTW** | | | **After IPTW** | | | **Before IPTW** | | | **After IPTW** | | | **Before IPTW** | | | **After IPTW** | | |
|  |  | **VKA** | **Dab** | **STD*** | **VKA** | **Dab** | **STD*** | **VKA** | **Riv** | **STD*** | **VKA** | **Riv** | **STD*** | **VKA** | **Api** | **STD*** | **VKA** | **Api** | **STD*** |
| **Comedications (continued)** | |  |  |  |  |  |  |  |  |  |  |  |  |  |  |  |  |  |  |
| Oral corticosteroids | | 12.1 | 11.4 | -0.02 | 11.9 | 11.5 | -0.01 | 12.1 | 11.6 | -0.02 | 11.9 | 11.9 | 0.00 | 12.1 | 11.7 | -0.01 | 12.0 | 12.0 | 0.00 |
| Opioids and other analgesics | | 52.9 | 49.4 | -0.07 | 52.2 | 53.1 | 0.02 | 52.9 | 47.7 | -0.10 | 51.4 | 52.4 | 0.02 | 52.9 | 50.4 | -0.05 | 52.4 | 53.5 | 0.02 |
| Antiulcer agents | | 51.1 | 44.8 | -0.13 | 49.9 | 50.2 | 0.01 | 51.1 | 43.6 | -0.15 | 48.7 | 49.3 | 0.01 | 51.1 | 46.6 | -0.09 | 50.1 | 51.1 | 0.02 |
| Hypnotics or anxiolytics | | 29.6 | 25.3 | -0.10 | 28.8 | 29.3 | 0.01 | 29.6 | 23.8 | -0.13 | 27.9 | 28.6 | 0.02 | 29.6 | 24.2 | -0.12 | 28.4 | 29.2 | 0.02 |
| Homeopathy | | 28.7 | 29.3 | 0.01 | 28.8 | 28.7 | 0.00 | 28.7 | 30.5 | 0.04 | 29.3 | 29.4 | 0.00 | 28.7 | 33.3 | 0.10 | 29.8 | 30.3 | 0.01 |
| Polymedication (at index date) | |  |  |  |  |  |  |  |  |  |  |  |  |  |  |  |  |  |  |
|  | < 5 ATC classes | 40.3 | 59.9 | 0.40 | 44.1 | 45.8 | 0.03 | 40.3 | 62.7 | 0.46 | 47.4 | 48.2 | 0.02 | 40.3 | 58.5 | 0.37 | 44.3 | 45.4 | 0,02 |
|  | 5-9 ATC classes | 43.3 | 32.6 | -0.22 | 41.2 | 40.4 | -0.02 | 43.3 | 30.2 | -0.28 | 39.1 | 38.1 | -0.02 | 43.3 | 33.2 | -0.21 | 41.1 | 40.4 | -0,01 |
|  | ≥10 ATC classes | 16.4 | 7.5 | -0.28 | 14.7 | 13.8 | -0.02 | 16.4 | 7.1 | -0.29 | 13.5 | 13.6 | 0.00 | 16.4 | 8.4 | -0.24 | 14.6 | 14.2 | -0,01 |
| **Healthcare system use** | |  |  |  |  |  |  |  |  |  |  |  |  |  |  |  |  |  |  |
| First OAC prescriber’s specialty | |  |  |  |  |  |  |  |  |  |  |  |  |  |  |  |  |  |  |
|  | Hospital practitioner | 49.7 | 38.7 | -0.22 | 47.5 | 43.6 | -0.08 | 49.7 | 37.1 | -0.26 | 45.4 | 42.7 | -0.05 | 49.7 | 43.3 | -0.13 | 48.1 | 45.7 | -0,05 |
|  | General practitioner | 25.5 | 20.8 | -0.11 | 24.7 | 27.4 | 0.06 | 25.5 | 20.3 | -0.12 | 24.1 | 26.9 | 0.06 | 25.5 | 17.7 | -0.19 | 23.8 | 25.8 | 0,05 |
|  | Private cardiologist | 22.4 | 38.2 | 0.35 | 25.5 | 26.7 | 0.03 | 22.4 | 40.3 | 0.39 | 28.2 | 28.1 | 0.00 | 22.4 | 36.7 | 0.32 | 25.7 | 26.1 | 0,01 |
|  | Other private practitioners | 2.4 | 2.2 | -0.01 | 2.3 | 2.3 | 0.00 | 2.4 | 2.4 | 0.00 | 2.4 | 2.4 | 0.00 | 2.4 | 2.3 | -0.01 | 2.4 | 2.3 | 0,00 |
| General practitioner visits§ | |  |  |  |  |  |  |  |  |  |  |  |  |  |  |  |  |  |  |
|  | 0 | 3.2 | 3.1 | 0.00 | 3.2 | 3.0 | -0.01 | 3.2 | 3.4 | 0.01 | 3.2 | 3.1 | -0.01 | 3.2 | 3.0 | -0.01 | 3.1 | 2.9 | -0,01 |
|  | 1-5 | 31.9 | 36.0 | 0.09 | 32.6 | 31.3 | -0.03 | 31.9 | 38.7 | 0.14 | 33.8 | 32.5 | -0.03 | 31.9 | 37.8 | 0.12 | 33.1 | 32.1 | -0,02 |
|  | 6-11 | 40.1 | 40.2 | 0.00 | 40.1 | 40.5 | 0.01 | 40.1 | 39.3 | -0.02 | 39.9 | 39.9 | 0.00 | 40.1 | 40.0 | 0.00 | 40.1 | 40.2 | 0,00 |
|  | ≥12 | 24.8 | 20.6 | -0.10 | 24.1 | 25.1 | 0.03 | 24.8 | 18.7 | -0.15 | 23.0 | 24.6 | 0.04 | 24.8 | 19.2 | -0.14 | 23.6 | 24.8 | 0,03 |
| Influenza vaccination¶ | | 56.1 | 50.2 | -0.12 | 55.1 | 56.0 | 0.02 | 56.1 | 48.0 | -0.16 | 53.8 | 54.4 | 0.01 | 56.1 | 50.9 | -0.10 | 55.0 | 55.6 | 0.01 |

* An absolute standardized difference less than 0.1 was considered to be a negligible between-group difference.

† Comorbidities only defined by using diagnosis ICD-10 codes from hospital discharge and specific reimbursement status data.

‡ Smoking or alcoholism data: measured using proxies such as reimbursements for nicotine replacement therapy/drugs used in alcohol dependence and hospital discharge diagnoses related to tobacco use or alcohol abuse (see Supplementary Table 1).

§ Frequency of general practitioner visits was determined during the year before the index date.

¶ During the influenza vaccination campaign directly preceding the index date.

AF, atrial fibrillation; Api: apixaban; ATC, Anatomical Therapeutic Chemical; ATE: arterial thromboembolic events (ischaemic stroke, arterial systemic embolism or transient ischaemic attack); COPD: chronic obstructive pulmonary disease; Dab: dabigatran; DVT/PE: deep vein thrombosis/pulmonary embolism; IPTW: inverse probability of treatment weighting; NSAIDs: non-steroidal anti-inflammatory drugs; OAC: oral anticoagulant; Riv: rivaroxaban; VKA: vitamin K antagonist; SD: standard deviation; STD: standardized difference

## Supplementary Table 6. Baseline characteristics according to the type of OAC before and after inverse probability of treatment weighting in patients with prior liver disease

| **Characteristics**  **(column %, unless stated otherwise)** | | **Dabigatran *versus* VKA (N=1,120 *vs* 7,884)** | | | | | | **Rivaroxaban *versus* VKA (N=2,441 *vs* 7,884)** | | | | | | **Apixaban *versus* VKA (N=1,516 *vs* 7,884)** | | | | | |
| --- | --- | --- | --- | --- | --- | --- | --- | --- | --- | --- | --- | --- | --- | --- | --- | --- | --- | --- | --- |
|  |  | **Before IPTW** | | | **After IPTW** | | | **Before IPTW** | | | **Before IPTW** | | | **After IPTW** | | | **Before IPTW** | | |
|  |  | **VKA** | **Dab** | **STD*** | **VKA** | **Dab** | **STD*** | **VKA** | **Riv** | **STD*** | **VKA** | **Riv** | **STD*** | **VKA** | **Api** | **STD*** | **VKA** | **Api** | **STD*** |
| **Sociodemographic data** | |  |  |  |  |  |  |  |  |  |  |  |  |  |  |  |  |  |  |
| Age (years), mean  (SD) | | 72.0 (11.9) | 70.5 (11.6) | -0.13 | 71.9 (11.9) | 72.5 (12.0) | 0.05 | 72.0 (11.9) | 70.0 (11.8) | -0.18 | 71.6 (11.9) | 71.9 (11.8) | 0.02 | 72.0 (11.9) | 72.0 (11.8) | -0.01 | 72.0 (11.9) | 72.6 (11.6) | 0.05 |
|  | 18-54 | 7,9 | 9,6 | 0,06 | 8,1 | 7,1 | -0,04 | 7,9 | 11,1 | 0,11 | 8,6 | 8,3 | -0,01 | 7,9 | 8,3 | 0,01 | 8,0 | 7,3 | -0,03 |
|  | 55-64 | 18,7 | 19,0 | 0,01 | 18,7 | 17,7 | -0,03 | 18,7 | 19,0 | 0,01 | 18,7 | 18,1 | -0,01 | 18,7 | 17,0 | -0,04 | 18,4 | 17,6 | -0,02 |
|  | 65-74 | 26,9 | 31,5 | 0,10 | 27,5 | 26,9 | -0,01 | 26,9 | 31,7 | 0,10 | 28,1 | 28,4 | 0,01 | 26,9 | 30,0 | 0,07 | 27,5 | 27,5 | 0,00 |
|  | 75-79 | 16,1 | 14,9 | -0,03 | 15,9 | 17,3 | 0,04 | 16,1 | 15,1 | -0,03 | 15,9 | 14,9 | -0,03 | 16,1 | 14,3 | -0,05 | 15,8 | 15,1 | -0,02 |
|  | 80-84 | 14,9 | 14,4 | -0,02 | 14,8 | 13,8 | -0,03 | 14,9 | 13,2 | -0,05 | 14,5 | 16,0 | 0,04 | 14,9 | 15,6 | 0,02 | 15,0 | 16,0 | 0,03 |
|  | 85-89 | 10,7 | 7,9 | -0,10 | 10,4 | 12,3 | 0,06 | 10,7 | 7,3 | -0,12 | 9,9 | 9,3 | -0,02 | 10,7 | 10,5 | -0,01 | 10,7 | 11,7 | 0,03 |
|  | ≥90 | 4,8 | 2,7 | -0,11 | 4,5 | 4,9 | 0,02 | 4,8 | 2,7 | -0,11 | 4,3 | 4,8 | 0,03 | 4,8 | 4,4 | -0,02 | 4,7 | 4,9 | 0,01 |
| Female sex | | 40,7 | 42,4 | 0,03 | 40,9 | 39,7 | -0,02 | 40,7 | 40,0 | -0,01 | 40,4 | 40,1 | -0,01 | 40,7 | 41,7 | 0,02 | 40,8 | 40,8 | 0,00 |
| Deprivation index | |  |  |  |  |  |  |  |  |  |  |  |  |  |  |  |  |  |  |
|  | Quintile 1 (least deprived) | 15,3 | 16,1 | 0,02 | 15,4 | 17,0 | 0,04 | 15,3 | 17,9 | 0,07 | 15,8 | 15,6 | -0,01 | 15,3 | 15,4 | 0,00 | 15,3 | 15,5 | 0,01 |
|  | Quintile 2 | 18,3 | 18,8 | 0,01 | 18,4 | 20,1 | 0,04 | 18,3 | 17,7 | -0,02 | 18,2 | 19,0 | 0,02 | 18,3 | 18,9 | 0,01 | 18,4 | 18,9 | 0,01 |
|  | Quintile 3 | 18,8 | 19,3 | 0,01 | 18,9 | 18,7 | 0,00 | 18,8 | 18,7 | 0,00 | 18,7 | 17,1 | -0,04 | 18,8 | 18,8 | 0,00 | 18,8 | 17,8 | -0,03 |
|  | Quintile 4 | 21,0 | 22,1 | 0,03 | 21,1 | 19,7 | -0,03 | 21,0 | 21,2 | 0,00 | 21,1 | 20,5 | -0,01 | 21,0 | 20,2 | -0,02 | 20,9 | 21,5 | 0,01 |
|  | Quintile 5 (most deprived) | 24,7 | 21,5 | -0,08 | 24,3 | 21,9 | -0,06 | 24,7 | 23,0 | -0,04 | 24,3 | 26,0 | 0,04 | 24,7 | 24,9 | 0,00 | 24,7 | 24,4 | -0,01 |
|  | Overseas departments | 1,9 | 2,2 | 0,02 | 1,9 | 2,6 | 0,05 | 1,9 | 1,6 | -0,02 | 1,8 | 1,9 | 0,01 | 1,9 | 1,9 | 0,00 | 1,9 | 1,9 | 0,00 |
| **Comorbidities** | |  |  |  |  |  |  |  |  |  |  |  |  |  |  |  |  |  |  |
| Ischaemic heart diseases | | 36,5 | 28,0 | -0,18 | 35,5 | 34,9 | -0,01 | 36,5 | 28,8 | -0,16 | 34,7 | 33,7 | -0,02 | 36,5 | 31,5 | -0,11 | 35,7 | 35,1 | -0,01 |
| Vascular diseases | | 45,5 | 34,6 | -0,22 | 44,1 | 43,3 | -0,02 | 45,5 | 36,3 | -0,19 | 43,3 | 42,0 | -0,03 | 45,5 | 40,2 | -0,11 | 44,6 | 44,6 | 0,00 |
| Heart failure | | 49,6 | 38,1 | -0,23 | 48,3 | 52,2 | 0,08 | 49,6 | 39,7 | -0,20 | 47,3 | 48,0 | 0,01 | 49,6 | 45,4 | -0,09 | 49,0 | 51,7 | 0,05 |
| Arrhythmias (other than AF)† | | 31,6 | 26,5 | -0,11 | 31,0 | 34,4 | 0,07 | 31,6 | 28,6 | -0,06 | 31,0 | 31,4 | 0,01 | 31,6 | 29,9 | -0,04 | 31,3 | 31,1 | -0,01 |
| Diabetes | | 40,2 | 35,2 | -0,10 | 39,6 | 41,1 | 0,03 | 40,2 | 35,0 | -0,11 | 39,0 | 38,2 | -0,02 | 40,2 | 37,7 | -0,05 | 39,9 | 39,0 | -0,02 |

**Supplementary Table 6 (continued)**

| **Characteristics**  **(column %, unless stated otherwise)** | **Dabigatran *versus* VKA (N=1,120 *vs* 7,884)** | | | | | | **Rivaroxaban *versus* VKA (N=2,441 *vs* 7,884)** | | | | | | **Apixaban *versus* VKA (N=1,516 *vs* 7,884)** | | | | | |
| --- | --- | --- | --- | --- | --- | --- | --- | --- | --- | --- | --- | --- | --- | --- | --- | --- | --- | --- |
|  | **Before IPTW** | | | **After IPTW** | | | **Before IPTW** | | | **Before IPTW** | | | **After IPTW** | | | **Before IPTW** | | |
|  | **VKA** | **Dab** | **STD*** | **VKA** | **Dab** | **STD*** | **VKA** | **Riv** | **STD*** | **VKA** | **Riv** | **STD*** | **VKA** | **Api** | **STD*** | **VKA** | **Api** | **STD*** |
| **Comorbidities (continued)** |  |  |  |  |  |  |  |  |  |  |  |  |  |  |  |  |  |  |
| History of ATE† | 14,3 | 12,1 | -0,07 | 14,1 | 14,9 | 0,02 | 14,3 | 11,6 | -0,08 | 13,7 | 13,6 | 0,00 | 14,3 | 15,1 | 0,02 | 14,5 | 15,0 | 0,01 |
| Dementia or Parkinson’s disease | 9,9 | 6,5 | -0,12 | 9,5 | 11,8 | 0,07 | 9,9 | 6,7 | -0,11 | 9,1 | 10,3 | 0,04 | 9,9 | 9,0 | -0,03 | 9,7 | 10,5 | 0,02 |
| Epilepsy or mental illness | 31,3 | 32,1 | 0,02 | 31,4 | 34,1 | 0,06 | 31,3 | 31,3 | 0,00 | 31,4 | 32,4 | 0,02 | 31,3 | 31,3 | 0,00 | 31,3 | 32,0 | 0,01 |
| History of DVT/PE | 6,1 | 3,4 | -0,13 | 5,7 | 8,6 | 0,11 | 6,1 | 2,7 | -0,17 | 5,3 | 6,7 | 0,06 | 6,1 | 2,8 | -0,16 | 5,5 | 5,8 | 0,01 |
| Chronic kidney disease† | 23,5 | 9,8 | -0,37 | 21,9 | 25,4 | 0,08 | 23,5 | 9,3 | -0,39 | 20,2 | 21,1 | 0,02 | 23,5 | 12,1 | -0,30 | 21,7 | 20,3 | -0,03 |
| Asthma/COPD | 27,4 | 24,9 | -0,06 | 27,2 | 30,3 | 0,07 | 27,4 | 24,7 | -0,06 | 26,9 | 27,6 | 0,02 | 27,4 | 26,1 | -0,03 | 27,2 | 27,3 | 0,00 |
| History of bleeding† | 17,7 | 14,0 | -0,10 | 17,2 | 20,1 | 0,07 | 17,7 | 13,6 | -0,11 | 16,7 | 17,4 | 0,02 | 17,7 | 13,7 | -0,11 | 17,1 | 15,9 | -0,03 |
| Opioid-related disorders | 0,3 | 0,3 | -0,01 | 0,3 | 0,1 | -0,04 | 0,3 | 0,7 | 0,05 | 0,4 | 0,5 | 0,01 | 0,3 | 0,2 | -0,03 | 0,3 | 0,3 | -0,01 |
| Other chronic and debilitating diseases† | 11,9 | 10,5 | -0,04 | 11,7 | 11,2 | -0,02 | 11,9 | 12,0 | 0,00 | 12,0 | 13,4 | 0,04 | 11,9 | 11,9 | 0,00 | 11,9 | 11,5 | -0,01 |
| Frailty (proxy) | 35,0 | 22,5 | -0,28 | 33,5 | 35,1 | 0,03 | 35,0 | 23,7 | -0,25 | 32,3 | 34,4 | 0,04 | 35,0 | 28,0 | -0,15 | 33,9 | 35,3 | 0,03 |
| Thyroid diseases | 8,1 | 7,9 | -0,01 | 8,1 | 9,2 | 0,04 | 8,1 | 9,1 | 0,03 | 8,3 | 7,9 | -0,02 | 8,1 | 11,2 | 0,10 | 8,6 | 9,1 | 0,02 |
| Obesity† | 29,0 | 25,9 | -0,07 | 28,7 | 30,5 | 0,04 | 29,0 | 28,0 | -0,02 | 29,0 | 29,0 | 0,00 | 29,0 | 28,3 | -0,02 | 28,9 | 29,1 | 0,00 |
| Smoking‡ | 27,2 | 23,2 | -0,09 | 26,7 | 27,5 | 0,02 | 27,2 | 26,8 | -0,01 | 27,2 | 28,6 | 0,03 | 27,2 | 24,1 | -0,07 | 26,7 | 27,3 | 0,01 |
| **Comedications** |  |  |  |  |  |  |  |  |  |  |  |  |  |  |  |  |  |  |
| Potentially hepatotoxic drugs | 79,0 | 78,2 | -0,02 | 78,9 | 76,1 | -0,07 | 79,0 | 76,3 | -0,07 | 78,4 | 78,6 | 0,01 | 79,0 | 78,7 | -0,01 | 79,0 | 80,1 | 0,03 |
| Antihypertensive drugs | 91,6 | 90,6 | -0,03 | 91,5 | 91,0 | -0,02 | 91,6 | 87,5 | -0,13 | 90,7 | 91,0 | 0,01 | 91,6 | 89,1 | -0,08 | 91,2 | 91,1 | 0,00 |
| Antiarrhythmics or cardiac glycosides | 57,9 | 67,6 | 0,20 | 59,1 | 57,6 | -0,03 | 57,9 | 63,5 | 0,12 | 59,1 | 57,4 | -0,03 | 57,9 | 61,1 | 0,07 | 58,3 | 57,9 | -0,01 |
| Nitrovasodilator agents | 8,4 | 5,2 | -0,13 | 8,0 | 8,1 | 0,00 | 8,4 | 4,8 | -0,14 | 7,6 | 6,7 | -0,04 | 8,4 | 5,6 | -0,11 | 8,0 | 7,8 | -0,01 |
| Lipid-lowering agents | 42,8 | 40,4 | -0,05 | 42,5 | 42,5 | 0,00 | 42,8 | 40,0 | -0,06 | 42,2 | 41,3 | -0,02 | 42,8 | 44,7 | 0,04 | 43,2 | 43,8 | 0,01 |
| Antiplatelet drugs including aspirin | 52,4 | 48,6 | -0,08 | 52,0 | 51,8 | 0,00 | 52,4 | 49,2 | -0,07 | 51,8 | 51,8 | 0,00 | 52,4 | 49,4 | -0,06 | 52,0 | 51,8 | 0,00 |
| Parenteral anticoagulant (heparin) | 22,5 | 6,4 | -0,47 | 20,6 | 23,0 | 0,06 | 22,5 | 5,2 | -0,52 | 18,5 | 19,4 | 0,02 | 22,5 | 4,9 | -0,53 | 19,7 | 18,1 | -0,04 |
| NSAIDs or antirheumatic agents | 13,7 | 16,4 | 0,08 | 14,0 | 13,1 | -0,03 | 13,7 | 17,2 | 0,10 | 14,6 | 14,3 | -0,01 | 13,7 | 14,4 | 0,02 | 13,8 | 14,2 | 0,01 |

**Supplementary Table 6. (continued)**

| **Characteristics**  **(column %, unless stated otherwise)** | | **Dabigatran *versus* VKA (N=1,120 *vs* 7,884)** | | | | | | **Rivaroxaban *versus* VKA (N=2,441 *vs* 7,884)** | | | | | | **Apixaban *versus* VKA (N=1,516 *vs* 7,884)** | | | | | |
| --- | --- | --- | --- | --- | --- | --- | --- | --- | --- | --- | --- | --- | --- | --- | --- | --- | --- | --- | --- |
|  |  | **Before IPTW** | | | **After IPTW** | | | **Before IPTW** | | | **Before IPTW** | | | **After IPTW** | | | **Before IPTW** | | |
|  |  | **VKA** | **Dab** | **STD*** | **VKA** | **Dab** | **STD*** | **VKA** | **Riv** | **STD*** | **VKA** | **Riv** | **STD*** | **VKA** | **Api** | **STD*** | **VKA** | **Api** | **STD*** |
| **Comedications (continued)** | |  |  |  |  |  |  |  |  |  |  |  |  |  |  |  |  |  |  |
| Oral corticosteroids | | 13,7 | 13,5 | -0,01 | 13,7 | 13,4 | -0,01 | 13,7 | 12,3 | -0,04 | 13,4 | 12,7 | -0,02 | 13,7 | 14,1 | 0,01 | 13,8 | 13,5 | -0,01 |
| Opioids and other analgesics | | 55,5 | 52,6 | -0,06 | 55,1 | 53,7 | -0,03 | 55,5 | 55,8 | 0,01 | 55,8 | 57,7 | 0,04 | 55,5 | 55,5 | 0,00 | 55,5 | 55,9 | 0,01 |
| Antiulcer agents | | 63,6 | 57,9 | -0,12 | 62,9 | 64,5 | 0,03 | 63,6 | 54,6 | -0,18 | 61,6 | 61,5 | 0,00 | 63,6 | 59,7 | -0,08 | 63,0 | 63,0 | 0,00 |
| Hypnotics or anxiolytics | | 34,8 | 33,9 | -0,02 | 34,8 | 36,4 | 0,03 | 34,8 | 32,5 | -0,05 | 34,3 | 35,7 | 0,03 | 34,8 | 32,7 | -0,05 | 34,4 | 34,6 | 0,00 |
| Homeopathy | | 29,0 | 29,3 | 0,01 | 29,0 | 27,5 | -0,03 | 29,0 | 29,3 | 0,00 | 29,1 | 27,1 | -0,04 | 29,0 | 34,6 | 0,12 | 30,0 | 30,2 | 0,00 |
| Polymedication (at index date) | |  |  |  |  |  |  |  |  |  |  |  |  |  |  |  |  |  |  |
|  | < 5 ATC classes | 31,1 | 48,4 | 0,36 | 33,2 | 33,1 | 0,00 | 31,1 | 48,6 | 0,36 | 35,3 | 36,0 | 0,01 | 31,1 | 44,3 | 0,27 | 33,3 | 35,9 | 0,05 |
|  | 5-9 ATC classes | 44,3 | 37,2 | -0,15 | 43,5 | 46,0 | 0,05 | 44,3 | 37,4 | -0,14 | 42,7 | 42,7 | 0,00 | 44,3 | 37,7 | -0,13 | 43,3 | 42,4 | -0,02 |
|  | ≥10 ATC classes | 24,5 | 14,4 | -0,26 | 23,3 | 20,9 | -0,06 | 24,5 | 13,9 | -0,27 | 22,0 | 21,3 | -0,02 | 24,5 | 17,9 | -0,16 | 23,5 | 21,7 | -0,04 |
| **Health-care system use** | |  |  |  |  |  |  |  |  |  |  |  |  |  |  |  |  |  |  |
| First OAC prescriber’s specialty | |  |  |  |  |  |  |  |  |  |  |  |  |  |  |  |  |  |  |
|  | Hospital practitioner | 61,6 | 50,1 | -0,23 | 60,1 | 56,1 | -0,08 | 61,6 | 49,4 | -0,25 | 58,5 | 55,2 | -0,07 | 61,6 | 56,2 | -0,11 | 60,7 | 60,0 | -0,01 |
|  | General practitioner | 22,0 | 20,0 | -0,05 | 21,8 | 27,4 | 0,13 | 22,0 | 20,9 | -0,03 | 21,9 | 25,2 | 0,08 | 22,0 | 17,2 | -0,12 | 21,3 | 22,5 | 0,03 |
|  | Private cardiologist | 13,2 | 27,0 | 0,35 | 14,9 | 13,8 | -0,03 | 13,2 | 27,1 | 0,35 | 16,6 | 16,6 | 0,00 | 13,2 | 24,8 | 0,30 | 15,1 | 15,3 | 0,01 |
|  | Other private practictionner | 3,2 | 2,9 | -0,01 | 3,1 | 2,8 | -0,02 | 3,2 | 2,5 | -0,04 | 3,0 | 3,0 | 0,00 | 3,2 | 1,8 | -0,09 | 2,9 | 2,3 | -0,04 |
| General practitioner visit§ | |  |  |  |  |  |  |  |  |  |  |  |  |  |  |  |  |  |  |
|  | 0 | 4,0 | 2,5 | -0,08 | 3,8 | 3,6 | -0,01 | 4,0 | 3,1 | -0,05 | 3,7 | 3,2 | -0,03 | 4,0 | 3,2 | -0,04 | 3,8 | 3,2 | -0,03 |
|  | 1-5 | 28,3 | 29,4 | 0,02 | 28,4 | 26,6 | -0,04 | 28,3 | 28,8 | 0,01 | 28,4 | 28,0 | -0,01 | 28,3 | 31,0 | 0,06 | 28,8 | 29,6 | 0,02 |
|  | 6-11 | 36,5 | 39,0 | 0,05 | 36,8 | 35,3 | -0,03 | 36,5 | 40,8 | 0,09 | 37,6 | 37,6 | 0,00 | 36,5 | 37,8 | 0,03 | 36,7 | 35,0 | -0,04 |
|  | ≥12 | 31,1 | 29,1 | -0,04 | 30,9 | 34,5 | 0,08 | 31,1 | 27,3 | -0,08 | 30,4 | 31,2 | 0,02 | 31,1 | 28,0 | -0,07 | 30,7 | 32,2 | 0,03 |
| Influenza vaccination¶ | | 50,6 | 46,4 | -0,08 | 50,0 | 46,9 | -0,06 | 50,6 | 45,3 | -0,11 | 49,4 | 48,3 | -0,02 | 50,6 | 47,2 | -0,07 | 50,0 | 50,2 | 0,00 |

## Supplementary Table 7. Baseline characteristics according to the type of OAC before and after inverse probability of treatment weighting in patients in patients with history of alcohol abuse

| **Characteristics**  **(column %, unless stated otherwise)** | | **Dabigatran *versus* VKA (N=1,317 *vs* 7,779)** | | | | | | **Rivaroxaban *versus* VKA (N=2,524 *vs* 7,779)** | | | | | | **Apixaban *versus* VKA (N=1,553 *vs* 7,779)** | | | | | |
| --- | --- | --- | --- | --- | --- | --- | --- | --- | --- | --- | --- | --- | --- | --- | --- | --- | --- | --- | --- |
|  |  | **Before IPTW** | | | **After IPTW** | | | **Before IPTW** | | | **Before IPTW** | | | **After IPTW** | | | **Before IPTW** | | |
|  |  | **VKA** | **Dab** | **STD*** | **VKA** | **Dab** | **STD*** | **VKA** | **Riv** | **STD*** | **VKA** | **Riv** | **STD*** | **VKA** | **Api** | **STD*** | **VKA** | **Api** | **STD*** |
| **Sociodemographic data** | |  |  |  |  |  |  |  |  |  |  |  |  |  |  |  |  |  |  |
| Age (years), mean  (SD) | | 67.4 (11.2) | 66.1 (11.2) | -0.12 | 67.2 (11.2) | 67.8 (11.1) | 0.05 | 67.4 (11.2) | 65.6 (11.2) | -0.16 | 67.0 (11.2) | 67.4 (11.3) | 0.04 | 67.4 (11.2) | 66.4 (11.0) | -0.09 | 67.2 (11.2) | 67.7 (11.1) | 0.04 |
|  | 18-54 | 12,3 | 15,2 | 0,08 | 12,7 | 11,6 | -0,03 | 12,3 | 15,2 | 0,08 | 12,9 | 12,0 | -0,03 | 12,3 | 14,4 | 0,06 | 12,6 | 11,9 | -0,02 |
|  | 55-64 | 28,4 | 29,7 | 0,03 | 28,6 | 27,3 | -0,03 | 28,4 | 29,3 | 0,02 | 28,7 | 28,7 | 0,00 | 28,4 | 27,6 | -0,02 | 28,3 | 28,0 | -0,01 |
|  | 65-74 | 30,9 | 30,9 | 0,00 | 30,9 | 31,0 | 0,00 | 30,9 | 33,5 | 0,06 | 31,4 | 31,3 | 0,00 | 30,9 | 33,4 | 0,05 | 31,3 | 29,2 | -0,04 |
|  | 75-79 | 12,4 | 11,8 | -0,02 | 12,3 | 13,9 | 0,05 | 12,4 | 10,5 | -0,06 | 12,0 | 11,7 | -0,01 | 12,4 | 11,6 | -0,02 | 12,3 | 14,1 | 0,05 |
|  | 80-84 | 10,1 | 8,3 | -0,06 | 9,8 | 11,4 | 0,05 | 10,1 | 7,9 | -0,08 | 9,6 | 10,2 | 0,02 | 10,1 | 8,6 | -0,05 | 9,8 | 10,7 | 0,03 |
|  | 85-89 | 4,7 | 3,3 | -0,07 | 4,5 | 3,9 | -0,03 | 4,7 | 2,8 | -0,10 | 4,3 | 4,5 | 0,01 | 4,7 | 3,7 | -0,05 | 4,6 | 4,9 | 0,01 |
|  | ≥90 | 1,2 | 0,8 | -0,04 | 1,1 | 1,0 | -0,02 | 1,2 | 0,8 | -0,05 | 1,1 | 1,6 | 0,04 | 1,2 | 0,6 | -0,06 | 1,1 | 1,2 | 0,01 |
| Female sex | | 21,7 | 21,1 | -0,02 | 21,7 | 21,8 | 0,00 | 21,7 | 22,6 | 0,02 | 22,0 | 23,2 | 0,03 | 21,7 | 20,3 | -0,03 | 21,5 | 22,9 | 0,03 |
| Deprivation index | |  |  |  |  |  |  |  |  |  |  |  |  |  |  |  |  |  |  |
|  | Quintile 1 (least deprived) | 13,5 | 12,8 | -0,02 | 13,4 | 13,6 | 0,01 | 13,5 | 14,3 | 0,02 | 13,6 | 12,9 | -0,02 | 13,5 | 13,6 | 0,00 | 13,5 | 13,2 | -0,01 |
|  | Quintile 2 | 16,9 | 16,5 | -0,01 | 16,8 | 17,7 | 0,02 | 16,9 | 17,2 | 0,01 | 17,0 | 17,1 | 0,00 | 16,9 | 18,9 | 0,05 | 17,2 | 17,4 | 0,00 |
|  | Quintile 3 | 18,9 | 18,8 | 0,00 | 18,8 | 18,5 | -0,01 | 18,9 | 19,7 | 0,02 | 19,0 | 19,0 | 0,00 | 18,9 | 19,3 | 0,01 | 18,9 | 18,1 | -0,02 |
|  | Quintile 4 | 22,6 | 22,2 | -0,01 | 22,5 | 23,0 | 0,01 | 22,6 | 22,7 | 0,00 | 22,6 | 23,5 | 0,02 | 22,6 | 20,8 | -0,04 | 22,3 | 22,5 | 0,00 |
|  | Quintile 5 (most deprived) | 25,7 | 26,3 | 0,01 | 25,8 | 24,5 | -0,03 | 25,7 | 23,7 | -0,05 | 25,3 | 25,3 | 0,00 | 25,7 | 25,4 | -0,01 | 25,7 | 26,0 | 0,01 |
|  | Overseas departments | 2,5 | 3,5 | 0,06 | 2,6 | 2,8 | 0,01 | 2,5 | 2,4 | -0,01 | 2,4 | 2,2 | -0,01 | 2,5 | 2,1 | -0,02 | 2,4 | 2,9 | 0,03 |
| **Comorbidities** | |  |  |  |  |  |  |  |  |  |  |  |  |  |  |  |  |  |  |
| Prior liver disease | | 27,2 | 21,2 | -0,14 | 26,4 | 29,2 | 0,06 | 27,2 | 20,7 | -0,15 | 25,6 | 26,4 | 0,02 | 27,2 | 21,1 | -0,14 | 26,2 | 27,5 | 0,03 |
| Ischaemic heart diseases | | 32,0 | 26,1 | -0,13 | 31,2 | 32,9 | 0,04 | 32,0 | 25,1 | -0,15 | 30,4 | 31,3 | 0,02 | 32,0 | 26,8 | -0,11 | 31,2 | 31,5 | 0,01 |
| Vascular diseases | | 42,9 | 34,3 | -0,18 | 41,7 | 43,3 | 0,03 | 42,9 | 32,9 | -0,21 | 40,5 | 41,0 | 0,01 | 42,9 | 36,5 | -0,13 | 41,9 | 42,3 | 0,01 |
| Heart failure | | 49,8 | 43,1 | -0,13 | 48,9 | 50,6 | 0,03 | 49,8 | 43,1 | -0,14 | 48,3 | 49,9 | 0,03 | 49,8 | 50,8 | 0,02 | 49,9 | 49,7 | -0,01 |
| Arrhythmias (other than AF)† | | 28,0 | 24,5 | -0,08 | 27,5 | 29,5 | 0,04 | 28,0 | 24,8 | -0,07 | 27,3 | 27,7 | 0,01 | 28,0 | 28,2 | 0,00 | 28,1 | 28,9 | 0,02 |
| Diabetes | | 32,0 | 29,2 | -0,06 | 31,6 | 34,0 | 0,05 | 32,0 | 26,0 | -0,13 | 30,7 | 31,4 | 0,02 | 32,0 | 28,1 | -0,09 | 31,4 | 31,2 | 0,00 |

**Supplementary Table 7. (continued)**

| **Characteristics**  **(column %, unless stated otherwise)** | **Dabigatran *versus* VKA (N=1,317 *vs* 7,779)** | | | | | | **Rivaroxaban *versus* VKA (N=2,524 *vs* 7,779)** | | | | | | **Apixaban *versus* VKA (N=1,553 *vs* 7,779)** | | | | | |
| --- | --- | --- | --- | --- | --- | --- | --- | --- | --- | --- | --- | --- | --- | --- | --- | --- | --- | --- |
|  | **Before IPTW** | | | **After IPTW** | | | **Before IPTW** | | | **Before IPTW** | | | **After IPTW** | | | **Before IPTW** | | |
|  | **VKA** | **Dab** | **STD*** | **VKA** | **Dab** | **STD*** | **VKA** | **Riv** | **STD*** | **VKA** | **Riv** | **STD*** | **VKA** | **Api** | **STD*** | **VKA** | **Api** | **STD*** |
| Comorbidities (continued) |  |  |  |  |  |  |  |  |  |  |  |  |  |  |  |  |  |  |
| History of ATE† | 18,5 | 17,2 | -0,03 | 18,3 | 21,1 | 0,07 | 18,5 | 15,2 | -0,09 | 17,8 | 19,2 | 0,04 | 18,5 | 19,3 | 0,02 | 18,6 | 19,6 | 0,02 |
| Dementia or Parkinson’s disease | 9,3 | 7,8 | -0,05 | 9,1 | 10,5 | 0,05 | 9,3 | 7,0 | -0,08 | 8,8 | 9,7 | 0,03 | 9,3 | 7,5 | -0,06 | 9,0 | 10,9 | 0,06 |
| Epilepsy or mental illness | 36,0 | 37,4 | 0,03 | 36,2 | 38,9 | 0,06 | 36,0 | 38,2 | 0,05 | 36,5 | 37,7 | 0,02 | 36,0 | 34,7 | -0,03 | 35,8 | 37,4 | 0,03 |
| History of DVT/PE | 4,6 | 2,0 | -0,15 | 4,2 | 5,7 | 0,07 | 4,6 | 2,5 | -0,11 | 4,1 | 6,1 | 0,09 | 4,6 | 1,7 | -0,16 | 4,1 | 3,7 | -0,02 |
| Chronic kidney disease† | 14,3 | 5,0 | -0,32 | 13,0 | 15,5 | 0,07 | 14,3 | 5,7 | -0,29 | 12,2 | 12,2 | 0,00 | 14,3 | 7,5 | -0,22 | 13,2 | 14,7 | 0,04 |
| Asthma/COPD | 32,4 | 27,9 | -0,10 | 31,8 | 34,1 | 0,05 | 32,4 | 28,6 | -0,08 | 31,5 | 31,6 | 0,00 | 32,4 | 27,8 | -0,10 | 31,6 | 32,9 | 0,03 |
| History of bleeding† | 15,0 | 11,0 | -0,12 | 14,4 | 17,4 | 0,08 | 15,0 | 8,8 | -0,19 | 13,5 | 13,7 | 0,01 | 15,0 | 12,4 | -0,07 | 14,6 | 15,3 | 0,02 |
| Opioid-related disorders | 0,5 | 1,0 | 0,06 | 0,5 | 0,6 | 0,00 | 0,5 | 0,8 | 0,04 | 0,6 | 0,6 | 0,01 | 0,5 | 0,4 | -0,01 | 0,5 | 0,5 | 0,01 |
| Other chronic and debilitating diseases† | 7,7 | 7,1 | -0,02 | 7,6 | 7,4 | -0,01 | 7,7 | 7,0 | -0,03 | 7,6 | 8,1 | 0,02 | 7,7 | 6,3 | -0,06 | 7,5 | 8,1 | 0,02 |
| Frailty (proxy) | 36,1 | 27,0 | -0,20 | 34,8 | 36,0 | 0,03 | 36,1 | 28,4 | -0,17 | 34,3 | 37,1 | 0,06 | 36,1 | 29,6 | -0,14 | 35,1 | 38,8 | 0,08 |
| Thyroid diseases | 5,1 | 5,7 | 0,03 | 5,2 | 7,2 | 0,08 | 5,1 | 5,3 | 0,01 | 5,1 | 5,6 | 0,02 | 5,1 | 5,1 | 0,00 | 5,1 | 5,9 | 0,04 |
| Obesity† | 26,0 | 21,6 | -0,10 | 25,5 | 29,6 | 0,09 | 26,0 | 22,9 | -0,07 | 25,4 | 26,3 | 0,02 | 26,0 | 23,1 | -0,07 | 25,6 | 26,7 | 0,03 |
| Smoking‡ | 45,7 | 44,0 | -0,03 | 45,4 | 43,1 | -0,05 | 45,7 | 45,1 | -0,01 | 45,6 | 46,6 | 0,02 | 45,7 | 43,7 | -0,04 | 45,4 | 46,2 | 0,02 |
| **Comedications** |  |  |  |  |  |  |  |  |  |  |  |  |  |  |  |  |  |  |
| Potentially hepatotoxic drugs | 78,9 | 75,4 | -0,08 | 78,4 | 79,0 | 0,01 | 78,9 | 75,8 | -0,07 | 78,3 | 78,9 | 0,01 | 78,9 | 76,9 | -0,05 | 78,6 | 80,0 | 0,03 |
| Antihypertensive drugs | 89,8 | 87,9 | -0,06 | 89,5 | 90,1 | 0,02 | 89,8 | 85,7 | -0,12 | 88,8 | 89,9 | 0,03 | 89,8 | 87,3 | -0,08 | 89,4 | 89,9 | 0,02 |
| Antiarrhythmics or cardiac glycosides | 56,6 | 60,1 | 0,07 | 57,1 | 59,2 | 0,04 | 56,6 | 59,1 | 0,05 | 57,2 | 56,9 | 0,00 | 56,6 | 57,0 | 0,01 | 56,7 | 57,1 | 0,01 |
| Nitrovasodilator agents | 6,4 | 3,2 | -0,15 | 5,9 | 6,8 | 0,04 | 6,4 | 3,5 | -0,13 | 5,7 | 6,1 | 0,02 | 6,4 | 4,2 | -0,09 | 6,0 | 5,4 | -0,02 |
| Lipid-lowering agents | 44,7 | 44,2 | -0,01 | 44,7 | 45,4 | 0,01 | 44,7 | 40,4 | -0,09 | 43,9 | 44,4 | 0,01 | 44,7 | 46,2 | 0,03 | 45,0 | 44,8 | 0,00 |
| Antiplatelet drugs including aspirin | 46,8 | 43,7 | -0,06 | 46,4 | 49,8 | 0,07 | 46,8 | 41,4 | -0,11 | 45,7 | 47,5 | 0,04 | 46,8 | 43,3 | -0,07 | 46,3 | 48,3 | 0,04 |
| Parenteral anticoagulant (heparin) | 21,9 | 5,1 | -0,51 | 19,5 | 20,0 | 0,01 | 21,9 | 4,7 | -0,52 | 17,7 | 17,9 | 0,00 | 21,9 | 3,7 | -0,57 | 18,9 | 18,5 | -0,01 |
| NSAIDs or antirheumatic agents | 12,4 | 16,3 | 0,11 | 13,0 | 12,3 | -0,02 | 12,4 | 14,4 | 0,06 | 12,9 | 12,6 | -0,01 | 12,4 | 11,4 | -0,03 | 12,2 | 12,3 | 0,00 |

**Supplementary Table 7. (continued)**

| **Characteristics**  **(column %, unless stated otherwise)** | | **Dabigatran *versus* VKA (N=1,317 *vs* 7,779)** | | | | | | **Rivaroxaban *versus* VKA (N=2,524 *vs* 7,779)** | | | | | | **Apixaban *versus* VKA (N=1,553 *vs* 7,779)** | | | | | |
| --- | --- | --- | --- | --- | --- | --- | --- | --- | --- | --- | --- | --- | --- | --- | --- | --- | --- | --- | --- |
|  |  | **Before IPTW** | | | **After IPTW** | | | **Before IPTW** | | | **Before IPTW** | | | **After IPTW** | | | **Before IPTW** | | |
|  |  | **VKA** | **Dab** | **STD*** | **VKA** | **Dab** | **STD*** | **VKA** | **Riv** | **STD*** | **VKA** | **Riv** | **STD*** | **VKA** | **Api** | **STD*** | **VKA** | **Api** | **STD*** |
| **Comorbidities (continued)** | |  |  |  |  |  |  |  |  |  |  |  |  |  |  |  |  |  |  |
| Oral corticosteroids | | 10,8 | 10,5 | -0,01 | 10,7 | 10,4 | -0,01 | 10,8 | 10,6 | 0,00 | 10,7 | 10,5 | -0,01 | 10,8 | 10,5 | -0,01 | 10,7 | 11,0 | 0,01 |
| Opioids and other analgesics | | 46,2 | 46,5 | 0,01 | 46,3 | 48,9 | 0,05 | 46,2 | 45,2 | -0,02 | 46,1 | 48,0 | 0,04 | 46,2 | 46,5 | 0,01 | 46,2 | 47,6 | 0,03 |
| Antiulcer agents | | 58,0 | 51,8 | -0,13 | 57,2 | 60,2 | 0,06 | 58,0 | 50,2 | -0,16 | 56,2 | 56,8 | 0,01 | 58,0 | 52,9 | -0,10 | 57,2 | 57,5 | 0,01 |
| Hypnotics or anxiolytics | | 37,2 | 37,2 | 0,00 | 37,2 | 36,8 | -0,01 | 37,2 | 39,0 | 0,04 | 37,6 | 38,1 | 0,01 | 37,2 | 36,6 | -0,01 | 37,1 | 38,1 | 0,02 |
| Homeopathy | | 21,7 | 23,1 | 0,03 | 21,9 | 21,3 | -0,01 | 21,7 | 24,1 | 0,06 | 22,4 | 22,6 | 0,01 | 21,7 | 25,8 | 0,09 | 22,4 | 24,0 | 0,04 |
| Polymedication (at index date) | |  |  |  |  |  |  |  |  |  |  |  |  |  |  |  |  |  |  |
|  | < 5 ATC classes | 25,2 | 38,7 | 0,29 | 27,1 | 27,2 | 0,00 | 25,2 | 38,7 | 0,29 | 28,4 | 28,3 | 0,00 | 25,2 | 36,8 | 0,25 | 27,1 | 29,2 | 0,05 |
|  | 5-9 ATC classes | 50,8 | 45,6 | -0,10 | 50,0 | 49,1 | -0,02 | 50,8 | 46,7 | -0,08 | 49,8 | 49,3 | -0,01 | 50,8 | 46,6 | -0,08 | 50,1 | 49,3 | -0,02 |
|  | ≥10 ATC classes | 24,1 | 15,7 | -0,21 | 22,9 | 23,7 | 0,02 | 24,1 | 14,5 | -0,24 | 21,8 | 22,4 | 0,02 | 24,1 | 16,6 | -0,19 | 22,9 | 21,5 | -0,03 |
| **Health-care system use** | |  |  |  |  |  |  |  |  |  |  |  |  |  |  |  |  |  |  |
| First OAC prescriber’s specialty | |  |  |  |  |  |  |  |  |  |  |  |  |  |  |  |  |  |  |
|  | Hospital practitioner | 66,5 | 62,9 | -0,08 | 66,0 | 65,8 | 0,00 | 66,5 | 61,8 | -0,10 | 65,1 | 62,3 | -0,06 | 66,5 | 65,5 | -0,02 | 66,3 | 62,6 | -0,08 |
|  | General practitioner | 21,8 | 18,1 | -0,09 | 21,3 | 21,6 | 0,01 | 21,8 | 18,0 | -0,10 | 21,0 | 23,6 | 0,06 | 21,8 | 17,4 | -0,11 | 21,1 | 23,1 | 0,05 |
|  | Private cardiologist | 8,9 | 16,3 | 0,22 | 10,0 | 10,1 | 0,00 | 8,9 | 17,6 | 0,26 | 11,2 | 11,4 | 0,01 | 8,9 | 14,5 | 0,17 | 9,9 | 10,9 | 0,03 |
|  | Other private practictionner | 2,7 | 2,7 | 0,00 | 2,7 | 2,4 | -0,02 | 2,7 | 2,7 | 0,00 | 2,7 | 2,6 | 0,00 | 2,7 | 2,6 | -0,01 | 2,7 | 3,5 | 0,04 |
| General practitioner visit§ | |  |  |  |  |  |  |  |  |  |  |  |  |  |  |  |  |  |  |
|  | 0 | 5,6 | 4,8 | -0,04 | 5,5 | 4,9 | -0,03 | 5,6 | 5,0 | -0,03 | 5,4 | 4,6 | -0,03 | 5,6 | 5,9 | 0,01 | 5,6 | 4,7 | -0,04 |
|  | 1-5 | 35,7 | 38,4 | 0,06 | 36,1 | 35,1 | -0,02 | 35,7 | 39,7 | 0,08 | 36,5 | 35,6 | -0,02 | 35,7 | 40,6 | 0,10 | 36,6 | 37,6 | 0,02 |
|  | 6-11 | 34,1 | 34,5 | 0,01 | 34,2 | 32,8 | -0,03 | 34,1 | 32,3 | -0,04 | 33,6 | 33,1 | -0,01 | 34,1 | 33,4 | -0,02 | 34,0 | 32,0 | -0,04 |
|  | ≥12 | 24,6 | 22,3 | -0,05 | 24,3 | 27,2 | 0,07 | 24,6 | 23,1 | -0,03 | 24,4 | 26,7 | 0,05 | 24,6 | 20,2 | -0,11 | 23,9 | 25,7 | 0,04 |
| Influenza vaccination¶ | | 36,5 | 33,0 | -0,07 | 36,0 | 36,2 | 0,00 | 36,5 | 30,1 | -0,14 | 35,1 | 36,3 | 0,03 | 36,5 | 32,8 | -0,08 | 35,9 | 34,8 | -0,02 |

* An absolute standardized difference less than 0.1 was considered to be a negligible between-group difference.

† Comorbidities only defined by using diagnosis ICD-10 codes from hospital discharge and specific reimbursement status data.

‡ Smoking or alcoholism data: measured using proxies such as reimbursements for nicotine replacement therapy/drugs used in alcohol dependence and hospital discharge diagnoses related to tobacco use or alcohol abuse (see Supplementary Table 1).

§ Frequency of general practitioner visits was determined during the year before the index date.

¶ During the influenza vaccination campaign directly preceding the index date.

AF, atrial fibrillation; Api: apixaban; ATC, Anatomical Therapeutic Chemical; ATE: arterial thromboembolic events (ischaemic stroke, arterial systemic embolism or transient ischaemic attack); COPD: chronic obstructive pulmonary disease; Dab: dabigatran; DVT/PE: deep vein thrombosis/pulmonary embolism; IPTW: inverse probability of treatment weighting; NSAIDs: non-steroidal anti-inflammatory drugs; OAC: oral anticoagulant; Riv: rivaroxaban; VKA: vitamin K antagonist; SD: standard deviation; STD: standardized difference

## Supplementary Table 8. Acute liver injury outcome in patients with nonvalvular atrial fibrillation and no prior liver disease: number of patients by ICD-10 diagnostic code and patients with liver transplantation.

| **Codes** | **Code labels** | **Oral anticoagulant groups (main population)** | | | | | | | | | |
| --- | --- | --- | --- | --- | --- | --- | --- | --- | --- | --- | --- |
|  |  | **VKA** | | **Dabigatran** | | **Rivaroxaban** | | **apixaxaban** | | **Total** | |
|  |  | **N** | **%** | **N** | **%** | **N** | **%** | **N** | **%** | **N** | **%** |
| **ICD-10 code** |  |  |  |  | |  | |  | |  |  |
| K71.0 | Toxic liver disease with cholestasis | 22 | 18.6 | 3 | 11.5 | 3 | 6.5 | 5 | 17.2 | 33 | 15.2 |
| K71.1 | Toxic liver disease with hepatic necrosis | 3 | 2.6 |  |  | 3 | 6.5 | 1 | 3.4 | 7 | 3.2 |
| K71.2 | Toxic liver disease with acute hepatitis | 17 | 14.5 | 2 | 7.7 | 4 | 8.7 | 7 | 24.1 | 30 | 13.8 |
| K71.6 | Toxic liver disease with hepatitis not elsewhere classified | 3 | 2.6 | 1 | 3.8 | 5 | 10.9 |  |  | 9 | 4.1 |
| K71.9 | Toxic liver disease unspecified | 3 | 2.6 | 1 | 3.8 | 1 | 2.2 |  |  | 5 | 2.3 |
| K72.0 | Acute and subacute hepatic failure | 33 | 28.2 | 6 | 23.1 | 14 | 30.4 | 10 | 34.5 | 63 | 28.9 |
| K72.9 | Hepatic failure, unspecified | 6 | 5.1 | 7 | 26.9 | 6 | 13.0 | 2 | 6.9 | 21 | 9.6 |
| K75.2 | Nonspecific reactive hepatitis | 1 | 0.9 | 1 | 3.8 | 1 | 2.2 | 1 | 3.4 | 4 | 1.8 |
| K75.9 | Inflammatory liver disease, unspecified | 3 | 2.6 |  |  | 2 | 4.3 |  |  | 5 | 2.3 |
| R17 | Unspecified jaundice | 25 | 21.4 | 4 | 15.4 | 7 | 15.2 | 3 | 10.3 | 39 | 17.9 |
| **CCAM code** |  |  | |  | |  | |  |  |  |  |
| HLEA002, HLEA001 | Liver transplantation | 1 | 0.9 | 1 | 3.8 |  |  |  |  | 2 | 0.9 |
| **Total** |  | **117** | **100%** | **26** | **100%** | **46** | **100%** | **29** | **100%** | **218** | **100%** |

## Supplementary Table 9. Subgroup and sensitivity analyses: number of events and one-year cumulative incidences of hospitalized acute liver injury for each type of OAC for each type of NOAC, compared to VKA, in patients with no prior liver disease (main study population)

9. A. Dabigatran *versus* VKA

| **Type of analysis** | | **N patients**  *dabigatran vs VKA* | **Outcome** | **N events**  *dabigatran vs VKA* | **Crude one-year cumulative incidence with 95% CI (per 10,000):** *dabigatran vs VKA* | **Crude HR**  **with 95% CI** | **HR after IPTW**  **with 95% CI** |
| --- | --- | --- | --- | --- | --- | --- | --- |
| **Subgroups analyses** | |  |  |  |  |  |  |
| **ITT** | **≥ 80 years** | 18,938 vs 105,571 | Hospitalized ALI | 13 vs 55 | 7.2 (4.2-12.4) vs 5.6 (4.3-7.3) | 1.28 (0.70-2.34) | 1.09 (0.56-2.11) |
|  |  |  | Ancillary outcome | 550 vs 3,311 | 303.2 (279.2-329.2) vs 338.3 (327.1-349.8) | 0.90 (0.82-0.98) | 0.96 (0.87-1.05) |
| **ITT** | **Men** | 25,140 vs 102,256 | Hospitalized ALI | 13 vs 51 | 5.3 (3.1-9.1) vs 5.2 (4.0-6.9) | 1.01 (0.55-1.86) | 1.59 (0.93-2.72) |
|  |  |  | Ancillary outcome | 708 vs 3,904 | 342.9 (322.6-364.6) vs 398.3 (386.2-410.7) | 0.72 (0.66-0.78 | 0.86 (0.80-0.93 |
| **ITT** | **Women** | 26,597 vs 118,111 | Hospitalized ALI | 13 vs 66 | 5.0 (2.9-8.6) vs 5.9 (4.6-7.5) | 0.85 (0.47-1.54) | 0.89 (0.49-1.62) |
|  |  |  | Ancillary outcome | 645 vs 3,633 | 247.9 (229.7-267.6) vs 322.9 (312.7-333.4) | 0.77 (0.70-0.83) | 0.84 (0.78-0.91) |
| **ITT** | **No hepatotoxic drug**  **at baseline^*^** | 12,276 vs 42,726 | Hospitalized ALI | 6 vs 19 | 5.0 (2.2-11.1) vs 4.6 (2.9-7.2) | 1.08 (0.43-2.69) | 1.58 (0.68-3.69) |
|  |  |  | Ancillary outcome | 236 vs 1,150 | 195.4 (172.2-221.7) vs 280.0 (264.5-296.5) | 0.70 (0.60-0.80) | 0.75 (0.65-0.86 |
| **ITT** | **NOAC standard dose†** | 16,458 vs 220,367 | Hospitalized ALI | 5 vs 177 | 3.1 (1.3-7.3) vs 5.6 (4.7-6.7) | 0.55 (0.22-1.34) | 0.43 (0.14-1.37) |
|  |  |  | Ancillary outcome | 374 vs 7,537 | 228.7 (206.9-252.8) vs 358.0 (350.2-366.1) | 0.64 (0.57-0.70) | 0.77 (0.69-0.86) |
| **ITT** | **NOAC reduced dose†** | 35,279 vs 220,367 | Hospitalized ALI | 21 vs 117 | 6.1 (4.0-9.4) vs 5.6 (4.7-6.7) | 1.10 (0.69-1.75) | 1.25 (0.80-1.96) |
|  |  |  | Ancillary outcome | 979 vs 7,537 | 285.7 (268.6-303.9) vs 358.0 (350.2-366.1) | 0.79 (0.74-0.85) | 0.87 (0.82-0.93 |
| **ITT** | **HAS-BLED score ≥3** | 10,557 vs 96,295 | Hospitalized ALI | 13 vs 66 | 12.9 (7.5-22.2) vs 7.3 (5.7-9.3) | 1.75 (0.97-3.18) | 1.56 (0.83-2.92) |
|  |  |  | Ancillary outcome | 324 vs 3,948 | 319.4 (286.9-355.5) vs 436.4 (423.3-450.0) | 0.73 (0.65-0.81) | 0.80 (0.72-0.89) |
| **ITT** | **Obesity** | 5,978 vs 31,999 | Hospitalized ALI | 5 vs 25 | 8.7 (3.6-20.8) vs 8.3 (5.6-12.2) | 1.04 (0.40-2.71) | 1.35 (0.56-3.27) |
|  |  |  | Ancillary outcome | 148 vs 1,271 | 252.9 (215.6-296.4) vs 415.7 (393.8-438.6) | 0.60 (0.51-0.71) | 0.73 (0.62-0.85) |
| **ITT** | **Frailty (proxy)** | 7,112 vs 54,829 | Hospitalized ALI | 5 vs 43 | 7.6 (3.2-18.4) vs 8.7 (6.4-11.7) | 0.87 (0.34-2.19) | 1.56 (0.83-2.92) |
|  |  |  | Ancillary outcome | 207 vs 1,973 | 313.4 (274.0-358.4) vs 399.2 (382.3-416.9) | 0.78 (0.68-0.90) | 0.75 (0.65-0.87) |
| **Sensitivity analyses** | |  |  |  |  |  |  |
| **ITT** | **Adjustment on alcoholism^‡^** | 51,737 vs 220,367 | Hospitalized ALI | 26 vs 117 | 5.1 (3.5-7.5) vs 5.6 (4.7-6.7) | 0.92 (0.60-1.41) | 1.21 (0.82-1.80) |
|  |  |  | Ancillary outcome | 1,353 vs 7,537 | 267.3 (253.6-281.7) vs 358.0 (350.2-366.1) | 0.74 (0.70-0.79) | 0.85 (0.81-0.90 |
| **ITT** | **IPTW 0,5% truncation^§^** | 51,737 vs 220,367 | Hospitalized ALI | 26 vs 117 | 5.1 (3.5-7.5) vs 5.6 (4.7-6.7) | 0.92 (0.60-1.41) | 1.23 (0.83-1.84) |
|  |  |  | Ancillary outcome | 1,353 vs 7,537 | 267.3 (253.6-281.7) vs 358.0 (350.2-366.1) | 0.74 (0.70-0.79) | 0.85 (0.80-0.90) |
| **ITT** | **IPTW 1% truncation** | 51,737 vs 220,367 | Hospitalized ALI | 26 vs 117 | 5.1 (3.5-7.5) vs 5.6 (4.7-6.7) | 0.92 (0.60-1.41) | 1.21 (0.80-1.83) |
|  |  |  | Ancillary outcome | 1,353 vs 7,537 | 267.3 (253.6-281.7) vs 358.0 (350.2-366.1) | 0.74 (0.70-0.79) | 0.83 (0.78-0.88) |
| **ITT** | **Asymmetric PS trimming** | 45,885 vs 178,541 | Hospitalized ALI | 26 vs 92 | 5.8 (3.9-8.5) vs 5.4 (4.4-6.6) | 1.08 (0,70-1.67) | 1.46 (0.97-2.21) |
|  |  |  | Ancillary outcome | 1,203 vs 5,788 | 268.0 (253.4-283.3) vs 337.7 (329.3-346.4) | 0.79 (0.74-0.84) | 0.87 (0.82-0.92) |
| **ITT** | **Outcome restriction 1^¶^** | 51,737 vs 220,367 | Hospitalized ALI | 22 vs 94 | 4.3 (2.9-6.6) vs 4.5 (3.7-5.5) | 0.97 (0.61-1.55) | 1.34 (0.87-2.04) |
|  |  |  | Ancillary outcome | NA | NA | NA | NA |
| **ITT** | **Outcome restriction 2^**^** | 51,737 vs 220,367 | Hospitalized ALI | 12 vs 79 | 2.4 (1.3-4.2) vs 3.8 (3.0-4.7) | 0.63 (0.34-1.16) | 0.79 (0.45-1.40) |
|  |  |  | Ancillary outcome | NA | NA | NA | NA |
| **Per-protocol analysis^††^** | | 51,737 vs 220,367 | Hospitalized ALI | 15 vs 99 | 3.5 (2.1-5.8) vs 5.4 (4.5-6.6) | 0.71 (0,41-1.21) | 1.02 (0.63-1.64) |
|  |  |  | Ancillary outcome | 913 vs 6,531 | 227.2 (212.8-242.7) vs 354.5 (346.0-363.1) | 0.65 (0.60-0.69) | 0.76 (0.72-0.82) |

**Supplementary Table 9. (continued)**

9. B. Rivaroxaban *versus* VKA

| **Type of analysis** | | **N patients**  *rivaroxaban vs VKA* | **Outcome** | **N events**  *rivaroxaban vs VKA* | **Crude one-year cumulative incidence with 95% CI (per 10,000):** *rivaroxaban vs VKA* | **Crude HR**  **with 95% CI** | **HR after IPTW**  **with 95% CI** |
| --- | --- | --- | --- | --- | --- | --- | --- |
| **Subgroups analyses** | |  |  |  |  |  |  |
| **ITT** | **≥ 80 years** | 32,665 *vs* 105,571 | Hospitalized ALI | 15 *vs* 55 | 4.8 (2.9-8.0) vs 5.6 (4.3-7.3) | 0.86 (0.48-1.51) | 1.31 (0.79-2.16) |
|  |  |  | Ancillary outcome | 1,008 vs 3,311 | 323.4 (304.3-343.6) vs 338.3 (327.1-349.8) | 0.95 (0.89-1.02) | 1.00 (0.93-1.07) |
| **ITT** | **Men** | 49,780 *vs* 102,256 | Hospitalized ALI | 23 *vs* 51 | 4.7 (3.1-7.1) vs 5.2 (4.0-6.9) | 0.90 (0.55-1.48) | 1.35 (0.85-2.15) |
|  |  |  | Ancillary outcome | 1,506 vs 3,904 | 308.4 (293.5-324.2) vs 398.3 (386.2-410.7) | 0.77 (0.73-0.82) | 0.95 (0.89-1.00) |
| **ITT** | **Women** | 49,628 *vs* 118 111 | Hospitalized ALI | 23 *vs* 66 | 4.7 (3.2-7.1) vs 5.9 (4.6-7.5) | 0.81 (0.50-1.29) | 1.43 (0.96-2.14) |
|  |  |  | Ancillary outcome | 1,371 vs 3,633 | 282.6 (268.2-297.7) vs322.9 (312.7-333.4) | 0.87 (0.82-0.93) | 0.99 (0.94-1.06) |
| **ITT** | **No hepatotoxic drug**  **at baseline‡** | 24,920 *vs* 42,726 | Hospitalized ALI | 8 *vs* 19 | 3.3 (1.6-6.5) vs 4.6 (2.9-7.2) | 0.70 (0.31-1.61) | 2.03 (1.03-4.02) |
|  |  |  | Ancillary outcome | 549 vs 1,150 | 223.6 (205.9-242.9) vs 280.0 (264.5-296.5) | 0.80 (0.72-0.88) | 0.97 (0.88-1.07) |
| **ITT** | **NOAC standard dose†** | 60,896 *vs* 220,367 | Hospitalized ALI | 26 *vs* 177 | 4.3 (3.0-6.4) vs 5.6 (4.7-6.7) | 0.77 (0.51-1.18) | 1.44 (1.01-2.04) |
|  |  |  | Ancillary outcome | 1,628 vs 7,537 | 270.3 (257.7-283.6) vs 358.0 (350.2-366.1) | 0.75 (0.71-0.79 | 0.95 (0.90-0.99) |
| **ITT** | **NOAC reduced dose†** | 38,512 *vs* 220,367 | Hospitalized ALI | 20 *vs* 117 | 5.4 (3.5-8.3) vs 5.6 (4.7-6.7) | 0.97 (0.60-1.56) | 1.29 (0.84-1.98) |
|  |  |  | Ancillary outcome | 1,249 vs 7,537 | 336.8 (318.9-355.6) vs 358.0 (350.2-366.1) | 0.94 (0.88-0.99) | 1.02 (0.97-1.09) |
| **ITT** | **HAS-BLED score ≥3** | 17,575 *vs* 96,295 | Hospitalized ALI | 14 *vs* 66 | 8.4 (5.0-14.1) vs 7.3 (5.7-9.3) | 1.14 (0.64-2.03) | 2.38 (1.54-3.67) |
|  |  |  | Ancillary outcome | 661 vs 3,948 | 392.5 (364.2-422.9) vs 436.4 (423.3-450.0) | 0.90 (0.82-0.97) | 0.93 (0.86-1.01) |
| **ITT** | **Obesity** | 11,611 *vs* 31,999 | Hospitalized ALI | 5 *vs* 25 | 4.4 (1.8-10.6) vs 8.3 (5.6-12.2) | 0.54 (0.31-1.40) | 1.72 (0.90-3.29) |
|  |  |  | Ancillary outcome | 408 vs 1,271 | 359.3 (326.6-395.2) vs 415.7 (393.8-438.6) | 0.86 (0.77-0.96) | 1.01 (0.91-1.13) |
| **ITT** | **Frailty (proxy)** | 13,204 *vs* 54,829 | Hospitalized ALI | 12 *vs* 43 | 9.9 (5.6-17.4) vs 8.7 (6.4-11.7) | 1.12 (0.59-2.13) | 1.47 (0.81-2.65) |
|  |  |  | Ancillary outcome | 500 vs 1,973 | 407.6 (374.0-444.2) vs 399.2 (382.3-416.9) | 1.02 (0.93-1.13) | 1.01 (0.92-1.12) |
| **Sensitivity analyses** | |  |  |  |  |  |  |
| **ITT** | **Adjustment on alcoholism^‡^** | 99,408 *vs* 220,367 | Hospitalized ALI | 46 vs 117 | 4.7 (3.6-6.3) vs 5.6 (4.7-6.7) | 0.85 (0.60-1.19) | 1.42 (1.05-1.92) |
|  |  |  | Ancillary outcome | 2,877 vs 7,5371 | 295.6 (285.1-306.4) vs 358.0 (350.2-366.1) | 0.82 (0.79-0.86) | 0.97 (0.93-1.01) |
| **ITT** | **IPTW 0.5% truncation^§^** | 99,408 *vs* 220,367 | Hospitalized ALI | 46 vs 117 | 4.7 (3.6-6.3) vs 5.6 (4.7-6.7) | 0.85 (0.60-1.19) | 1.24 (0.91-1.71) |
|  |  |  | Ancillary outcome | 2,877 vs 7,537 | 295.6 (285.1-306.4) vs 358.0 (350.2-366.1) | 0.82 (0.79-0.86) | 0.95 (0.91-0.99) |
| **ITT** | **IPTW 1% truncation** | 99,408 *vs* 220,367 | Hospitalized ALI | 46 vs 117 | 4.7 (3.6-6.3) vs 5.6 (4.7-6.7) | 0.85 (0.60-1.19) | 1.19 (0.86-1.64) |
|  |  |  | Ancillary outcome | 2,877 vs 7,537 | 295.6 (285.1-306.4) vs 358.0 (350.2-366.1) | 0.82 (0.79-0.86) | 0.94 (0.90-0.98) |
| **ITT** | **Asymmetric PS trimming** | 85,930 vs 178,620 | Hospitalized ALI | 39 vs 95 | 4.7 (3.4-6.4) vs 5.6 (4.5-6.8) | 0.83 (0.58-1.21) | 1.07 (0.74-1.54) |
|  |  |  | Ancillary outcome | 2,530 vs 5,880 | 300.8 (289.5-312.6) vs 343.2 (334.6-351.9) | 0.87 (0.83-0.92) | 0.95 (0.91-1.00) |
| **ITT** | **Outcome restriction 1^¶^** | 99,408 *vs* 220,367 | Hospitalized ALI | 39 vs 94 | 4.0 (2.9-5.5) vs 4.5 (3.7-5.5) | 0.90 (0.62-1.30) | 1.24 (0.88-1.77) |
|  |  |  | Ancillary outcome | NA | NA | NA | NA |
| **ITT** | **Outcome restriction 2^**^** | 99,408 *vs* 220,367 | Hospitalized ALI | 24 vs 79 | 2.5 (1.7-3.7) vs 3.8 (3.0-4.7) | 0.66 (0.42-1.04) | 0.76 (0.48-1.20) |
|  |  |  | Ancillary outcome | NA | NA | NA | NA |
| **Per-protocol analysis^††^** | | 99,408 *vs* 220,367 | Hospitalized ALI | 35 *vs* 99 | 4.6 (3.3-6.4) vs 5.4 (4.5-6.6) | 0.82 (0.56-1.21) | 1.44 (1.03-2.01) |
|  |  |  | Ancillary outcome | 2,102 vs 6,531 | 265.0 (253.9-276.7) vs 354.5 (346.0-363.1) | 0.74 (0.71-0.78) | 0.87 (0.82-0.93) |

**Supplementary Table 9. (continued)**

9. C. Apixaban *versus* VKA

| **Type of analysis** | | **N patients**  *apixaban vs VKA* | **Outcome** | **N events**  *apixaban vs VKA* | **Crude one-year cumulative incidence with 95% CI (per 10.000):** *apixaban vs VKA* | **Crude HR**  **with 95% CI** | **HR after IPTW**  **with 95% CI** |
| --- | --- | --- | --- | --- | --- | --- | --- |
| **Subgroups analyses** | |  |  |  |  |  |  |
| **ITT** | **≥ 80 years** | 25,400 *vs* 105,571 | Hospitalized ALI | 14 vs 55 | 5.8 (3.5-9.9) vs 5.6 (4.3-7.3) | 1.03 (0.57-1.85) | 1.11 (0.62-1.99) |
|  |  |  | Ancillary outcome | 846 vs 3 311 | 348.8 (326.5-372.7) vs 338.3 (327.1-349.8) | 1.03 (0.96-1.11) | 1.10 (1.02-1.18) |
| **ITT** | **Men** | 29,495 *vs* 102,256 | Hospitalized ALI | 13 vs 51 | 4.5 (2.6-7.8) vs5.2 (4.0-6.9) | 0.87 (0.47-1.59) | 0.59 (0.29-1.24) |
|  |  |  | Ancillary outcome | 990 vs 3,904 | 342.9 (322.6-364.6) vs 398.3 (386.2-410.7) | 0.86 (0.80-0.92) | 1.00 (0.93-1.07) |
| **ITT** | **Women** | 33,008 *vs* 118,111 | Hospitalized ALI | 16 vs 66 | 5.0 (3.1-8.2) vs 5.9 (4.6-7.5) | 0.84 (0.49-1.46) | 0.95 (0.56-1.62) |
|  |  |  | Ancillary outcome | 968 vs 3,633 | 300.7 (282.6-320.0) vs 322.9 (312.7-333.4) | 0.93 (0.87-1.00) | 1.02 (0.96-1.10) |
| **ITT** | **No hepatotoxic drug**  **at baseline^‡^** | 14,790 *vs* 42,726 | Hospitalized ALI | 5 vs 19 | 3.5 (1.5-8.4) vs 4.6 (2.9-7.2) | 0.74 (0.28-1.99) | 0.62 (0.20-1.90) |
|  |  |  | Ancillary outcome | 360 vs 1,150 | 247.8 (223.7-274.3) vs 280.0 (264.5-296.5) | 0.88 (0.79-1.00) | 0.95 (0.85-1.07) |
| **ITT** | **NOAC standard dose†** | 37,920 *vs* 220,367 | Hospitalized ALI | 17 vs 177 | 4.5 (2.8-7.3) vs 5.6 (4.7-6.7) | 0.81 (0.49-1.35) | 1.23 (0.79-1.91) |
|  |  |  | Ancillary outcome | 1,083 vs 7,537 | 288.7 (272.3-306.2) vs 358.0 (350.2-366.1) | 0.80 (0.75-0.86) | 0.98 (0.93-1.05) |
| **ITT** | **NOAC reduced dose†** | 24,583 *vs* 220,367 | Hospitalized ALI | 12 vs 117 | 5.1 (2.9-9.1) vs 5.6 (4.7-6.7) | 0.92 (0.51-1.66) | 0.51 (0.23-1.13) |
|  |  |  | Ancillary outcome | 875 vs 7,537 | 371.8 (348.3-396.8) vs 358.0 (350.2-366.1) | 1.04 (0.97-1.11) | 1.13 (1.05-1.21) |
| **ITT** | **HAS-BLED score ≥3** | 13,620 *vs* 96,295 | Hospitalized ALI | 5 vs 66 | 3.8 (1.6-9.1) vs 7.3 (5.7-9.3) | 0.53 (0.21-1.30) | 0.85 (0.81-0.90) |
|  |  |  | Ancillary outcome | 556 vs 3,948 | 425.9 (392.6-462.0) vs 436.4 (423.3-450.0) | 0.97 (0.89-1.06) | 1.09 (1.00-1.19) |
| **ITT** | **Obesity** | 7,784 *vs* 31,999 | Hospitalized ALI | 0 vs 25 | NA vs 8.3 (5.6-12.2) | NA | NA |
|  |  |  | Ancillary outcome | 267 vs 1,271 | 351.4 (312.3-395.3) vs 415.7 (393.8-438.6) | 0.84 (0.74--0.96) | 1.03 (0.91-1.17) |
| **ITT** | **Frailty (proxy)** | 10,561 *vs* 54,829 | Hospitalized ALI | 6 vs 43 | 6.0 (2.7-13.4) vs 8.7 (6.4-11.7) | 0.70 (0.30-1.65) | 0.66 (0.27-1.62) |
|  |  |  | Ancillary outcome | 407 vs 1,973 | 412.7 (375.2-453.9) vs 399.2 (382.3-416.9) | 1.04 (0.93-1.15) | 1.12 (1.01-1.25) |
| **Sensitivity analyses** | |  |  |  |  |  |  |
| **ITT** | **Adjustment on alcoholism^‡^** | 62,503 vs 220,367 | Hospitalized ALI | 29 vs 117 | 4.8 (3.3-6.9) vs 5.6 (4.7-6.7) | 0.85 (0.57-1.28) | 0.82 (0.54-1.26) |
|  |  |  | Ancillary outcome | 958 vs 7,537 | 320.7 (307.0-335.0) vs 358.0 (350.2-366.1) | 0.89 (0.85-0.94) | 1.01 (0.96-1.06) |
| **ITT** | **IPTW 0.5% truncation^§^** | 62,503 vs 220,367 | Hospitalized ALI | 29 vs 117 | 4.8 (3.3-6.9) vs 5.6 (4.7-6.7) | 0.85 (0.57-1.28) | 0.79 (0.51-1.23) |
|  |  |  | Ancillary outcome | 1,958 vs 7,537 | 320.7 (307.0-335.0) vs 358.0 (350.2-366.1) | 0.89 (0.85-0.94) | 1.00 (0.95-1.05) |
| **ITT** | **IPTW 1% truncation** | 62,503 vs 220,367 | Hospitalized ALI | 29 vs 117 | 4.8 (3.3-6.9) vs 5.6 (4.7-6.7) | 0.85 (0.57-1.28) | 0.78 (0.50-1.23) |
|  |  |  | Ancillary outcome | 1,958 vs 7,537 | 320.7 (307.0-335.0) vs 358.0 (350.2-366.1) | 0.89 (0.85-0.94) | 0.99 (0.94-1.04) |
| **ITT** | **Asymmetric PS trimming** | 55,232 vs 174,822 | Hospitalized ALI | 27 vs 98 | 5.0 (3.4-7.3) vs 5.9 (4.8-7.2) | 0.85 (0.56-1.31) | 0.71 (0.44-1.16) |
|  |  |  | Ancillary outcome | 1,762 vs 5,740 | 327.0 (312.3-342.3) vs 343.6 (335.0-352.4) | 0.95 (0.90-1.00) | 1.02 (0.97-1.08) |
| **ITT** | **Outcome restriction 1^¶^** | 62,503 vs 220,367 | Hospitalized ALI | 26 vs 94 | 4.3 (2.9-6.3) vs 4.5 (3.7-5.5) | 0.95 (0.62-1.47) | 0.88 (0.55-1.39) |
|  |  |  | Ancillary outcome | NA | NA | NA | NA |
| **ITT** | **Outcome restriction 2^**^** | 62,503 vs 220,367 | Hospitalized ALI | 23 vs 79 | 3.8 (2.5-5.7) vs 3.8 (3.0-4.7) | 1.00 (0.63-1.60) | 0.96 (0.59-1.56) |
|  |  |  | Ancillary outcome | NA | NA | NA | NA |
| **Per-protocol analysis^††^** | | 62,503 vs 220,367 | Hospitalized ALI | 22 vs 99 | 4.2 (2.8-6.4) vs 5.4 (4.5-6.6) | 0.77 (0.48-1.21) | 0.81 (0.51-1.29) |
|  |  |  | Ancillary outcome | 1,595 vs 6,531 | 299.5 (285.2-314.5) vs 354.5 | 0.84 (0.80-0.89) | 0.95 (0.90-1.00) |

* The list of drugs considered to be potentially hepatotoxic was provided in Supplementary Table 2.

† Standard doses: dabigatran 150 mg. rivaroxaban 20 mg and apixaban 5 mg; Reduced doses: dabigatran 110 or 75 mg. rivaroxaban 15 or 10 mg and apixaban 2.5mg.

‡ Further adjustment on history of alcohol abuse measured using proxies (see Supplementary Table 1).

§ In the IPTW method, all weights with value above the 99.9th percentile and below the 0.1th percentile were set equal to the 99.9th and the 0.1th percentiles, respectively.

¶ The list of ICD-10 codes used for outcome definition in the main analysis was restricted to acute or unspecified toxic liver disease codes (K71.0, K71.1, K71.2, K71.6, K71.8, K71.9, K72.0, K72.9, K75.2 or K75.9) or hepatic transplantation.

** The list of ICD-10 codes used for outcome definition in the main analysis was further restricted to acute toxic liver disease codes (K71.0, K71.1, K71.2 or K72.0) or hepatic transplantation only

†† Follow-up was censored at treatment discontinuation or switch to another oral anticoagulant.

CI: confidence interval; IPTW: inverse probability of treatment weighting; ITT: intention-to-treat analysis; NOAC: non-vitamin K antagonist oral anticoagulant; VKA: vitamin K antagonist.

## Supplementary Figure 2. Cumulative incidence of hospitalized acute liver injury by type of oral anticoagulant up to one year after treatment initiation in patients with no prior liver disease (main study population)

The proportional hazards assumption was checked by testing for correlation of the scaled Schoenfeld residuals with time (*Therneau TM, Grambsch PM. Modeling Survival Data: Extending the Cox Model. 1st ed. Springer-Verlag New York 2000*) and there was no evidence against proportional hazards.

### Dabigatran versus VKA


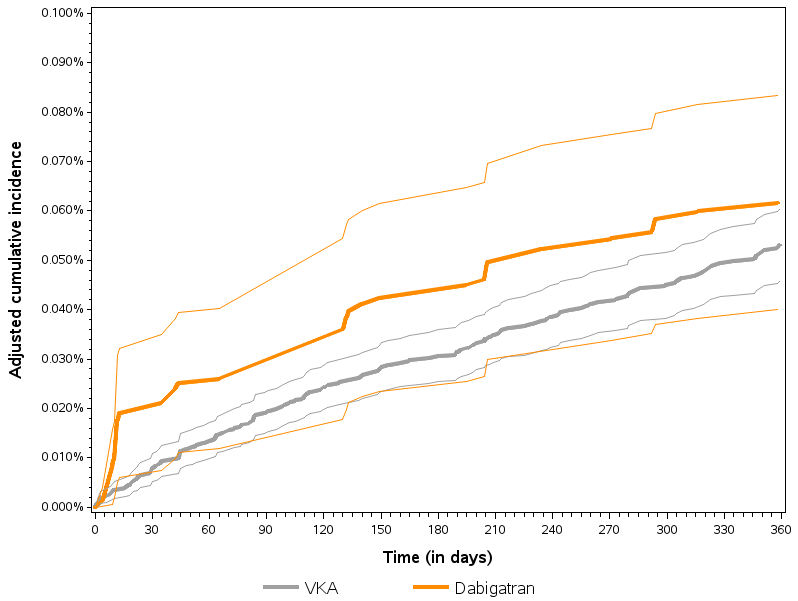

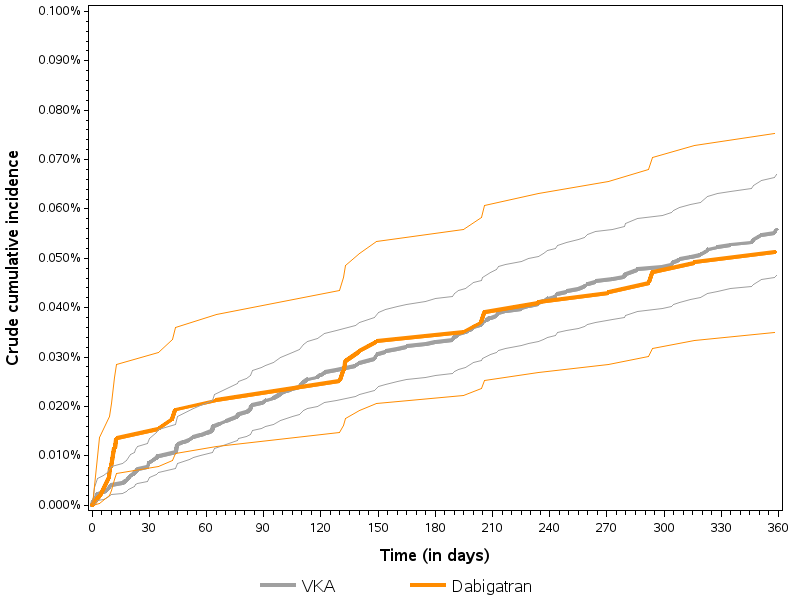


### Rivaroxaban versus VKA


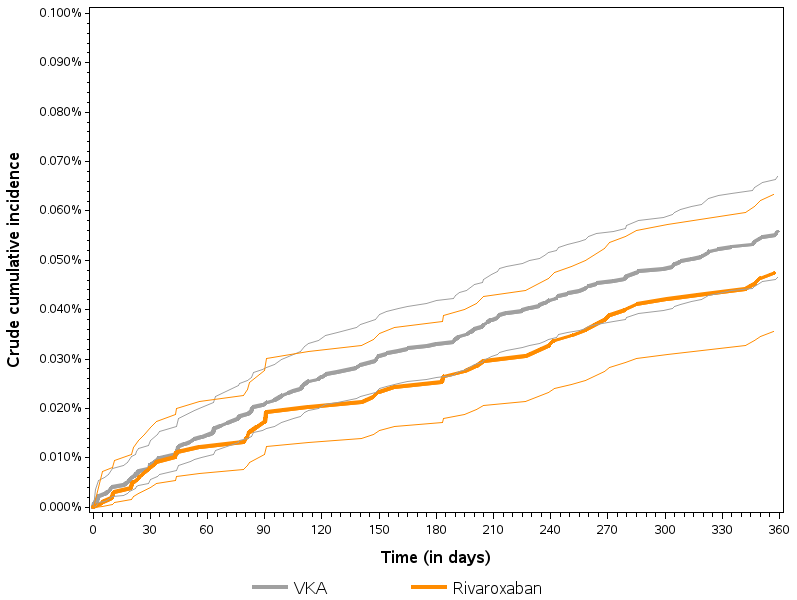

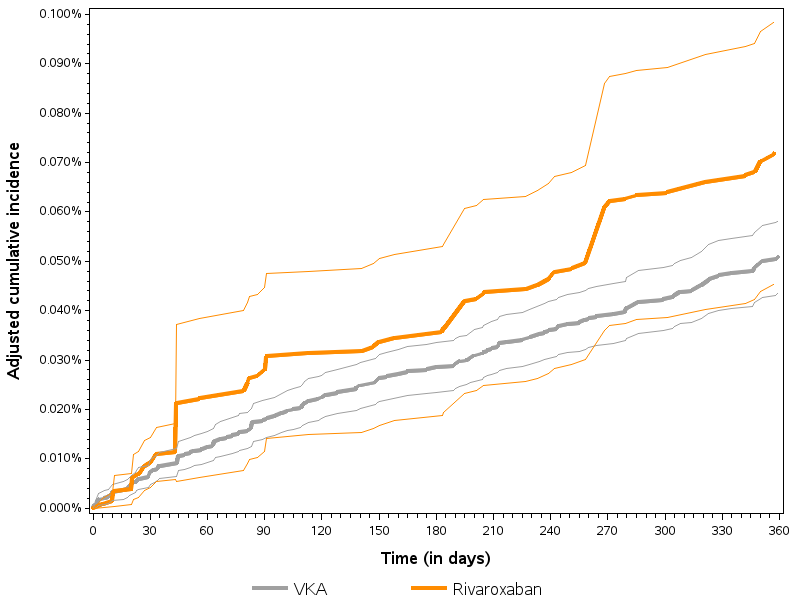


### Apixaban versus VKA


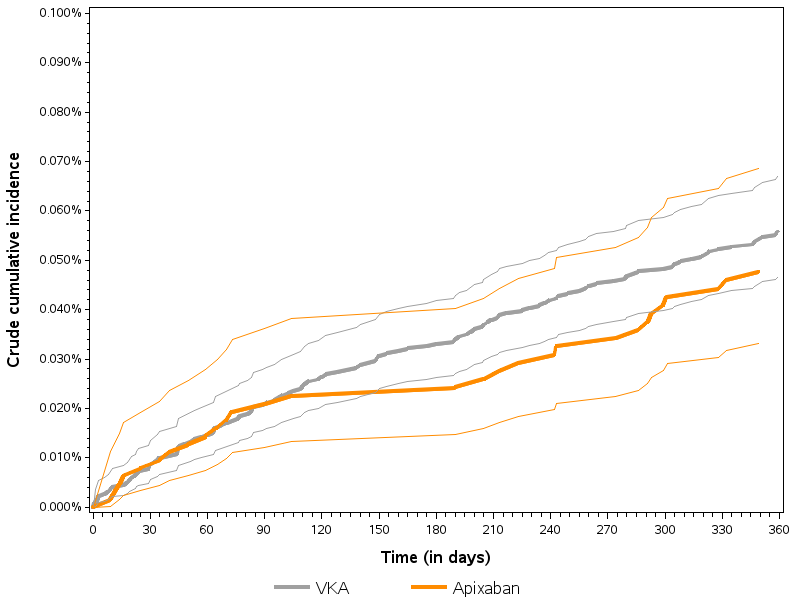

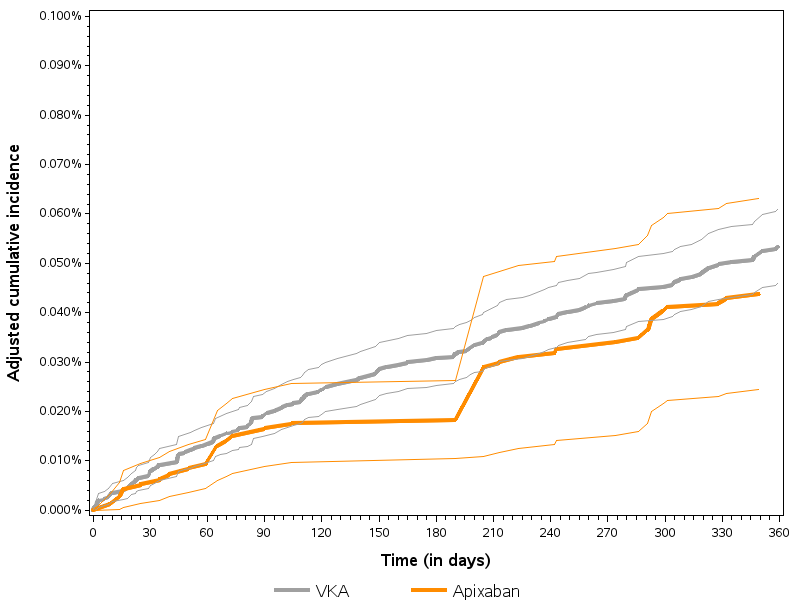

Supplement: Supplementary file 1 — Supplementary information. [file 41598_2020_68304_MOESM1_ESM.docx]
